# Recent Advances in Crimean-Congo Hemorrhagic Fever Virus Detection, Treatment, and Vaccination: Overview of Current Status and Challenges

**DOI:** 10.1186/s12575-024-00244-3

**Published:** 2024-06-26

**Authors:** Khursheed Muzammil, Saba Rayyani, Ahmed Abbas Sahib, Omid Gholizadeh, Hayder Naji Sameer, Tareq Jwad Kazem, Haneen Badran Mohammed, Hesam Ghafouri Kalajahi, Rahadian Zainul, Saman Yasamineh

**Affiliations:** 1 Department of Public Health, College of Applied Medical Sciences, King Khalid University, Khamis Mushait Campus, Abha, 62561 Saudi Arabia; 2 Medical Faculty, University of Georgi, Tbilisi, Georgia; 3 Department of Pharmacy, Mazaya University College, Dhi Qar, Iraq; 4 Azad Researcher, Virology and Biotechnology, Tehran, Iran; 5 Collage of Pharmacy, National University of Science and Technology, Dhi Qar, 64001 Iraq; 6 Scientific Affairs Department, Al-Mustaqbal University, Hillah, Babylon, 51001 Iraq; 7 Optics techniques department, health and medical techniques college, Al-Noor University, Mosul, Iraq; 8 Department of Biotechnology, Institute of Science, Uskudar University, Istanbul, Turkey; 9 Department of Chemistry, Faculty of Mathematics and Natural Sciences, Universitas Negeri Padang, Padang, Indonesia; 10 Center for Advanced Material Processing, Artificial Intelligence, and Biophysics Informatics (CAMPBIOTICS), Universitas Negeri Padang, Padang, Indonesia

**Keywords:** Crimean-Congo hemorrhagic fever virus (CCHFV), Detection, Treatment, Vaccination

## Abstract

Crimean-Congo hemorrhagic fever virus (CCHFV) is a tick-borne virus, and zoonosis, and affects large regions of Asia, Southwestern and Southeastern Europe, and Africa. CCHFV can produce symptoms, including no specific clinical symptoms, mild to severe clinical symptoms, or deadly infections. Virus isolation attempts, antigen-capture enzyme-linked immunosorbent assay (ELISA), and reverse transcription polymerase chain reaction (RT-PCR) are all possible diagnostic tests for CCHFV. Furthermore, an efficient, quick, and cheap technology, including biosensors, must be designed and developed to detect CCHFV. The goal of this article is to offer an overview of modern laboratory tests available as well as other innovative detection methods such as biosensors for CCHFV, as well as the benefits and limits of the assays. Furthermore, confirmed cases of CCHF are managed with symptomatic assistance and general supportive care. This study examined the various treatment modalities, as well as their respective limitations and developments, including immunotherapy and antivirals. Recent biotechnology advancements and the availability of suitable animal models have accelerated the development of CCHF vaccines by a substantial margin. We examined a range of potential vaccines for CCHF in this research, comprising nucleic acid, viral particles, inactivated, and multi-epitope vaccines, as well as the present obstacles and developments in this field. Thus, the purpose of this review is to present a comprehensive summary of the endeavors dedicated to advancing various diagnostic, therapeutic, and preventive strategies for CCHF infection in anticipation of forthcoming hazards.

**Graphical Abstract:**

**Figure:**
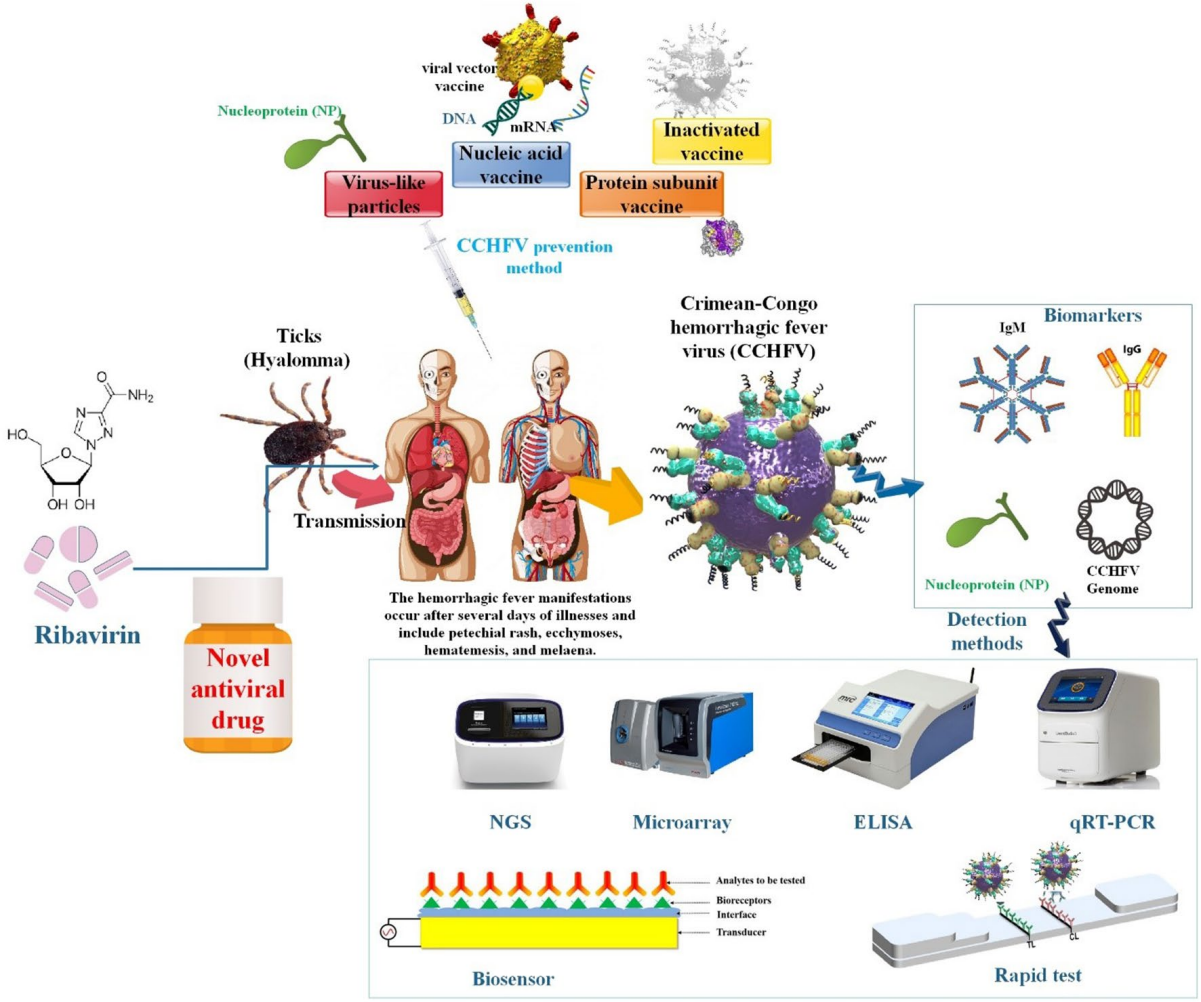
For the protection of medical personnel and effective case management, an early diagnosis of Crimean-Congo hemorrhagic fever (CCHF) is critical. CCHF is diagnosed through laboratory procedures such as RT-PCR, ELISA, virus isolation attempts, and ELISA detection of IgG and IgM antibodies. This review examines several biomarkers researched for their potential use in the diagnosis and prognosis of critical viral infections. It also explores the utility of more traditional diagnostic markers in predicting secondary complications, distinguishing Crimean-Congo hemorrhagic fever virus (CCHFV) infection, and serving as severity indicators. CCHFV vaccine development is advancing at an accelerated rate, facilitated by the availability of a lethal animal infection model. Hence, this review aims to furnish a comprehensive synopsis of the endeavors devoted to various vaccine candidates utilizing distinct approaches against CCHFV. These candidates comprise inactivated, virus-like particles, recombinant proteins, and nucleic acid vaccines. Furthermore, supportive therapy serves as the principal modality of treatment. Human cases of CCHF have been treated with ribavirin, a broad-spectrum antiviral medication; nevertheless, the therapeutic advantages of this intervention remain elusive. This article analyzes the present advancements and prospective trajectories in the realm of antiviral approaches targeting CCHFV.

## Introduction

**Fig. 1:**
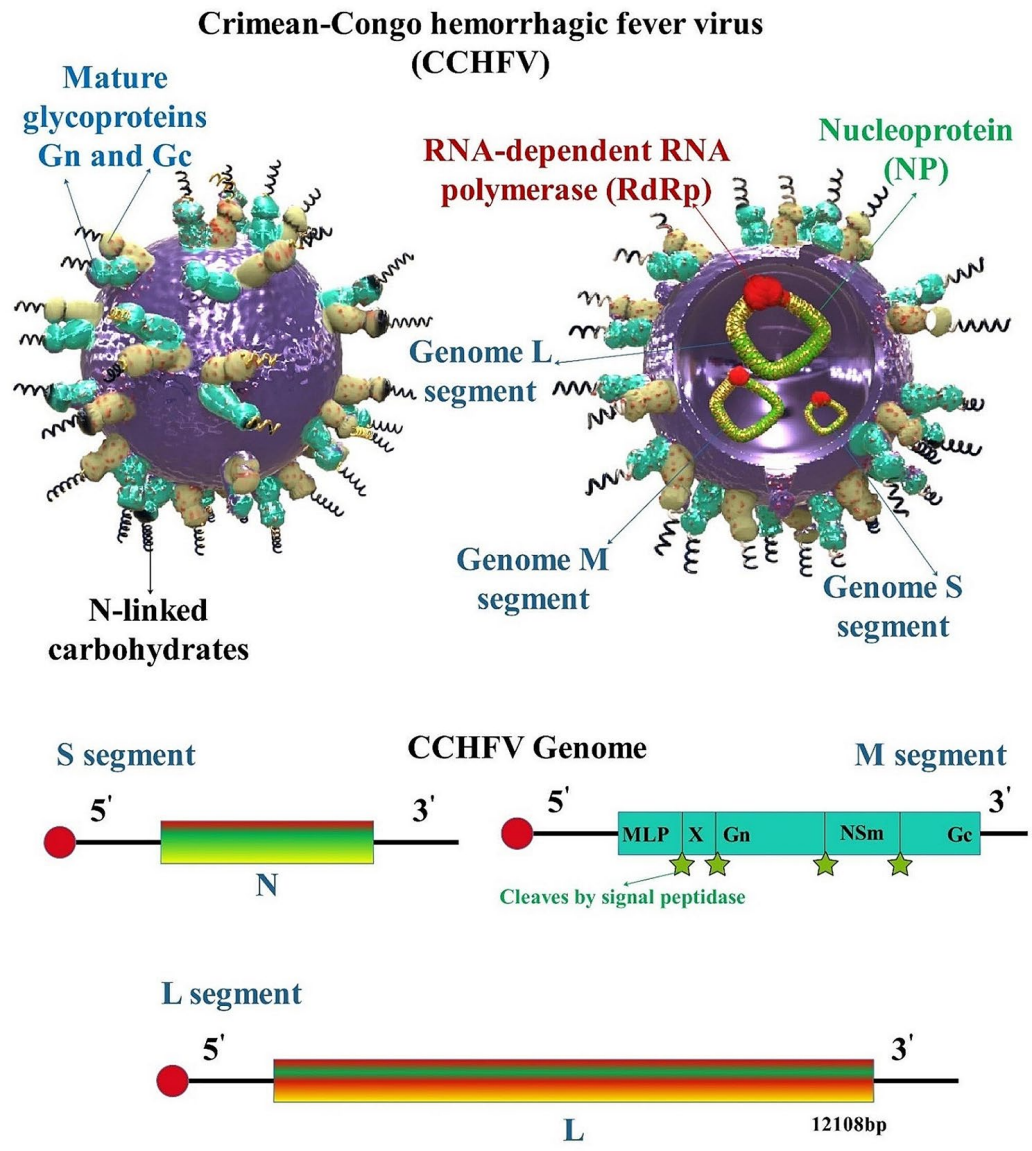
Structure of CCHFV and genomic structure. The negative-stranded RNA linear genome is made up of three segments: L, M, and S. Each segment of structural proteins that are implicated in viral life cycle processes such as assembly, replication, encapsidation, immunological evasion, and transit carries genetic information. These structural proteins comprise the polyprotein and ribonucleoprotein complex: RNA polymerase, nucleoprotein precursors (GPC), and NP. The M segment is responsible for generating a combination of wholly or partially cleaved intracellular proteins known as Precursor glycoprotein N-terminal (Pre-Gn), non-structural M protein (NSM), and Precursor glycoprotein C-terminal (Pre-Gc). Conversely, the S segment encodes the non-structural S protein (NSS), expressed at low levels and rapidly degraded by the proteasome in CCHFV-infected cell lines [22]

Crimean–Congo hemorrhagic fever virus (CCHFV) evidence was first recorded in the former Soviet Union’s Crimean region in 1944, and numerous other infected persons were documented in other southern Soviet republics, South Africa, and Bulgaria over the next years. Because the same disease was discovered in the Congo area in 1969, CCHFV was labeled “Crimean-Congo hemorrhagic fever” [1]. CCHFV is a global tick-borne infection with a high death rate (30% or more in certain cases) and a broad geographic range. Cases of CCHF, which is caused by CCHFV, have been recorded from Africa to Middle East Asia to Southern and Eastern Europe. No licensed vaccines or specialized antivirals exist to treat CCHF, and as the Hyalomma tick’s distribution expands, so do the people at risk. Additionally, despite yearly reports of CCHF, knowledge of the host and viral drivers of CCHFV pathogenesis is limited. CCHFV’s relationship with both creatures and humans is dichotomous. Although CCHFV can infect a wide variety of domesticated and feral mammalian species, including ovines and bovines, as well as specific avian species like ostriches, it does not induce substantial morbidity in these organisms [2, 3]. The World Health Organization (WHO) considers the CCHFV to be one of the seven highest prevalence infectious diseases of epidemic potential. Because of its epidemic potential, high lethality of human clinical infections, and lack of effective mitigation strategies, it poses a severe public health risk that requires immediate investigation [4, 5]. CCHFV is a negative-sense single-stranded-RNA virus with a negative sense (CCHFV; family *Nairoviridae*, genus *Orthonairovirus*) [6–9]. Among arboviruses, CCHFV has the largest genetic diversity; isolates differ in nucleotide sequences by 20% for the viral S segment and 31% for the viral M segment [10–12]. The Crimean Congo virus is enveloped and spherical with a size of 90 nm and a negative single-strand RNA virus with three fragments. CCHFV is a negative-sense, tri-segmented RNA bunyavirus member of the *Nairoviridae* family. CCHFV is characterized by a genome consisting of three parts, each containing negative polarity RNA. It is transmitted and maintained by ticks. The small (S), medium (M), and large (L) segments of CCHFV have non-coding regions (NCRs) at their 5′ and 3′ termini. These NCRs contribute to the circular appearance of the bunyavirus genomes. The nine terminal nucleotides (5′-UCUCAAAGA and 3′-AGAGUUUCU) are conserved among nairoviruses and act as viral promoter regions. The NCRs are essential for the viral RNA-dependent RNA polymerase (RdRp or L protein) to bind and initiate transcription and/or replication of the viral genome. Although the complete sequences of NCRs differ between viral segments, each NCR can initiate encapsidation, transcription, replication, and packaging of the genomes into new virions [13, 14]. Until recently, it was thought that each of the three segments of CCHFV encoded a single protein. However, it has been discovered that a second protein, the non-structural S (NSS), is encoded in the S segment in the opposite orientation relative to the nucleoprotein (NP) gene, suggesting that CCHFV may be an ambisense virus. The ambisense coding in CCHFV involves overlapping coding regions, which is different from ambisense coding in bunyaviruses and arenaviruses, where the viral proteins are encoded in the opposite orientation and separated by an intergenic region acting as a transcription termination signal. The S segment (~ 1.6 kb), which is similar to other bunyaviruses, is significantly larger than those of other bunyaviruses [15, 16]. Encapsidated viral RNA (vRNA) segments form genomic ribonucleoprotein (RNPs) complexes with NP and L proteins. The lipid envelope and the surface glycoproteins (GPs) Gn and Gc are acquired by the genomic RNPs, allowing them to be packed into viral particles [16]. As shown by its interactions with vRNA to form RNPs complexes, its endonuclease activity, its interactions with host heat shock proteins during intracellular viral replication and in infectious particles, and its promotion of viral mRNA translation, NP plays a variety of roles in the CCHFV life cycle. Controlling host cell apoptosis is essential for the CCHFV life cycle, as shown by the ability of NP and NSs to impact it. Both in vitro and in vivo, CCHFV infection results in the death of host cells, and it has been shown that those infected with the virus have higher levels of biomarkers linked to apoptosis. Therefore, it is unclear whether host apoptosis is pro- or antiviral. In vitro, when host apoptosis was inhibited, virus titers rose, suggesting an antiviral impact. CCHFV NP may inhibit the activation of caspase 3 and caspase 9, as well as the induction of apoptosis caused by BAX and the release of cytochrome c from mitochondria, despite the fact it is unclear where in the intrinsic apoptosis pathway NP stops activation. All of these results point to the possibility that CCHFV suppresses this host response via the usage of its NP protein and that the virus may be inhibited by host apoptosis [17]. Cleavage of the M polyprotein precursor generates the two envelope GPs, three secreted nonstructural proteins (GP38, GP85, or GP160, representing GP38 alone or GP38 linked to a mucin-like protein (MLD)), and a double-membrane-spanning protein known as NSm. Although the precise roles of these proteins remain unknown, it has been demonstrated that inhibiting GP38 expression and secretion correlates with a decline in preGn to Gn conversion and a slowing of growth kinetics in the early stages of an infection. Additionally, the CCHFV MLD-GP38 proteins (GP160/GP85) contain an N-terminal domain encompassing Furin-secreted MLD from GP38. Previously, the significance of GP38 in combating the lethal CCHFV challenge was underscored by the protective effect of non-neutralizing monoclonal antibodies (mAbs) that target GP38 [18–21] (Fig. 1).

The virus is mainly spread to humans through the bite of infected *Hyalomma spp*. ticks. Humans can contract CCHFV through contact with the blood, secretions, organs, or other bodily fluids of infected individuals and livestock during slaughter, in addition to infection via mosquito bites. This increases the susceptibility of individuals in intimate contact with animals, such as pastoralists, veterinarians, and abattoir staff, to CCHFV infections. The villagers, butcher, hospital staff, rancher, veterinarian, and others susceptible to tick bites are all at risk [23, 24]. Because CCHFV is challenging to detect in human hosts, it goes unrecognized until human cases emerge, distinguished by a fast onset of symptoms [25, 26]. The unprecedented rise in both expansion patterns and the quantity of CCHFV patients is because of the lack of adequate and appropriate epidemiologic studies in both animals and humans, which is related to a lack of inexpensive and efficacious diagnosis and therapy in prevalent regions of CCHFV [8]. Early diagnosis of CCHF is crucial for patient treatment and infection control. An efficient CCHF rapid test therefore is of great significance [27]. While viral isolation is the established method for diagnosing CCHF, its implementation is restricted to high-containment biosafety level 4 facilities, limiting the number of laboratories capable of employing this technique. In addition, viral isolation is not devoid of error or uncertainty because cell cultures lack sensitivity and typically only detect the relatively high viremia level observed during the initial five days of illness. Consequently, reference laboratories have been employing the most optimal and feasible techniques to ascertain the existence or nonexistence of an infection. For detection of the viral genome, these techniques include conventional and real-time quantitative reverse transcription PCR (RT-PCR and qRT-PCR). In contrast, for detection of specific IgM and IgG antibodies, they involve indirect immunofluorescence assays (IFAs) or ELISAs [28]. RT–PCR is the recommended instrument for fast laboratory detection in the acute disease state among the numerous diagnostic procedures [29–32]. As a result, while RT-PCR may be effective in the lab, it is challenging to apply in settings with limited resources and field hospitals. Commercially available serological diagnostic kits are used to detect IgM and IgG antibodies. But by the time a detectable Ab titer materializes, the patient has begun to heal, which lessens its significance for the patient’s clinical outcome. Owing to the high mortality rate (40%) it is critical to identify the infection in its early stages, i.e., on days 3–6, so that the patient can begin supportive treatment. Early on in an infection, antigen detection offers a simple substitute for virus detection. Although the CCHFV virus is extensively disseminated, antigen detection systems are not readily available globally. Thus, the creation of a secure ELISA assay is desperately needed to detect the CCHFV antigen in a variety of matrices, such as serum, ticks, and culture fluids. Due to its high conservation and immunogenicity, the CCHFV NP is employed as an early diagnostic marker. Researchers describe a double Ab-based antigen capture ELISA as a quick and accurate method for CCHFV detection in this work. There are now particular polyclonal and mAbs against NP that are used as detector and capture antibodies, respectively. The assay demonstrated the ability to identify viral NP in various matrices, such as culture supernatant, tick saliva, and human serum. The sandwich ELISA (sELISA) assay that was developed had a detection limit of 25 ng of purified antigen. Its detection limit, as determined by comparison with a real-time RT-PCR, was 1000 genome equivalents of CCHFV. Moreover, the assay’s sensitivity and specificity were 100% compared to a commercial kit that used gamma-irradiated CCHFV. With its high sensitivity and specificity, the recently created sELISA could be a helpful tool for identifying the CCHF virus in ticks, humans, and culture supernatants. The test will be helpful as an alternative tool for diagnosing acute infection and is adaptable for screening of large-scale samples in resource constraint situations [33].

In a different study, scientists developed a method to isolate the recombinant CCHFV NP, which is highly conserved antigenically across many CCHFV lineages and clades. They examined its potential use in an ELISA to identify antibodies specific to CCHFVs. Human embryonic kidney 293 T cells were transfected with the pCAGGS mammalian expression plasmid, which included the cloned NP gene. Using cesium-chloride gradient centrifugation, the expressed NP molecule was separated from the cell lysate. The antigen for the ELISA utilized to find anti-CCHFV IgG was purified NP. Researchers successfully identified CCHFV-specific IgG in anti-NP rabbit antiserum and CCHFV-infected monkey serum using the CCHFV NP-based ELISA. This test demonstrated comparable efficacy in identifying CCHFV-specific IgG in human sera compared to the commercially available Blackbox CCHFV IgG ELISA kit. These findings show that our CCHFV NP-based ELISA is a valuable tool for seroepidemiological investigations [34].

CCHFV is classified as a highly infectious virus that might result in a public health disaster and is thought to be an emergent arboviral zoonotic disease in many countries due to the absence of particular antiviral medicines, high fatality rate, increasing vector bionomics, and climate change. Therefore, it is imperative to discover innovative antiviral therapies against CCHFV to address the growing danger that CCHF poses to public health. At the moment, supportive therapy is the main course of treatment. Human instances of CCHF have been treated with ribavirin, a broad-spectrum antiviral drug; however, the therapeutic advantages are yet unclear. Additional treatment options used in case studies include particular immunoglobulin, convalescent serum, and steroids; however, insufficient data is available to evaluate these medications’ effectiveness. Furthermore, throughout the last several decades, prospective bunyavirus inhibitors have been studied, and some of them have shown promise in treating CCHFV infection [35, 36, 37]. Considering how long it takes to develop and approve antiviral medications, repurposing their usage for other ailments may be a tactic. There is a wealth of human dosage experience available for the majority of these medications. Furthermore, their distribution, metabolism, excretion, absorption, and safety characteristics are well established. It has been shown that the therapeutic medications chloroquine and chlorpromazine, which treat non-viral diseases, effectively suppresses CCHFV in vitro. The viral life cycle has numerous similar steps, including entrance, biosynthesis, assembly, and release, despite the variety of viruses. Broad-spectrum, host-directed antiviral medications often have great potential to target the comparable host processes and proteins that the virus makes use of. The virus mutation problem is a significant obstacle to the development of antiviral medications, particularly for RNA viruses like CCHFV have a high rate of mutation. Drug-resistant strains could emerge due to selective pressure from therapies that target viral components, which increases the likelihood of escape mutation. In contrast, host factor-directed intervention strategies would be more resistant to selective escape pressure and may have broad-spectrum antiviral potential. It should be highlighted; nevertheless, that host proteins often serve critical physiological roles for cells; as such, a more thorough assessment is required before targeting them for antiviral action. Conversely, viruses belonging to the same genus have similarities in their protein sequence and structure. Targets for developing pan-virus or pan-genus antivirals might include the catalytic domain of RNA polymerases, which is one of the essential viral proteins involved in viral infection. Since RdRp and OTU protease are prominent targets for drug design, most of the antivirals tested in the CCHFV case target these two proteins. However, inhibitors to other crucial and conserved viral components in the CCHFV life cycle must also be found and developed [37, 38]. The National Center for Infectious and Parasitic Diseases (BulBio-NCIPD Ltd.) in Bulgaria offers a preventative vaccination against CCHFV made from mouse brains and inactivated with chloroform. This vaccination was formerly mostly given to troops serving in rural areas. Nevertheless, regulated human trials and laboratory evaluations of the vaccine’s effectiveness are lacking, and the inactivated mouse brain-derived vaccine has not been scientifically shown to be protective against viral challenges in animal experiments. Furthermore, several nations prohibit the use of mouse brains in the creation of vaccines due to the possibility that autoimmune disorders like autoimmune encephalitis might manifest in people. Safe and more effective vaccinations are required to reduce CCHF in human populations because of the problems mentioned above and the unavailability of a safe vaccine for human usage [39]. Historically, a significant barrier to the development of vaccines and therapies has been the absence of an appropriate animal model that is sensitive to CCHFV infection. The discovery of vaccinations against CCHF has. However, it has been significantly accelerated by recent developments in biotechnology and the availability of appropriate animal models. These developments have aided in the assessment of possible vaccine candidates in addition to improving our knowledge of the pathophysiology of CCHF [40].

Currently, no vaccines have received approval, and the efficacy of antivirals like ribavirin remains uncertain. Therefore, the majority of treatment consists of supportive care. Leventhal et al. assessed an alphavirus-based replicon RNA vaccine, which expressed the glycoprotein or NP precursor of CCHFV, in a rigorous, heterologous lethal challenge mouse model in this report. Complete protection against clinical disease could be achieved through vaccination with RNA expressing the NP alone. However, for protection against disease and viral replication, vaccination with a combination of the NP and GPC provided robust protection. Protection against lethal challenge was achieved with a single 100ng of RNA vaccination. An unanticipated finding emerged from the examination of the immune responses induced by the components of the vaccine: vaccination-induced cellular immunity against the virion-exposed GP and antibodies against the internal viral NP [41].

With a focus on the diagnostic, preventative, and therapeutic potential of CCHFV in humans, this research offers a synopsis of current CCHFV prevalent and innovative approaches to diagnosis, treatment, and prevention. The future of these approaches, as well as their benefits and drawbacks, are also detailed.

## Crimean–Congo Hemorrhagic Fever Virus Life Cycle

Through clathrin-mediated endocytosis, which relies on the virus’s surface GPs, CCHFV penetrates the cell. It is uncertain, yet, which cellular receptors are necessary for CCHFV entrance. Researchers have shown that CCHFV enters the body via the low-density lipoprotein receptor (LDLR). In a variety of CCHFV-susceptible human, monkey, and mouse cells, genetic deletion of LDLR reduces viral infection. This is remedied upon reconstitution using ectopically produced LDLR. According to mutagenesis experiments, CCHFV infection requires the ligand binding domain (LBD) of LDLR. With great affinity, LDLR binds directly to the glycoprotein Gc of CCHFV, facilitating the virus’s attachment and internalization into host cells. Anti-LDLR blocking antibodies or a soluble sLDLR–Fc fusion protein consistently hinder CCHFV infection in various sensitive cells. Moreover, LDLR genetic deletion or LDLR-blocking Ab treatment dramatically lowers viral loads, pathogenic consequences, and mortality after CCHFV infection in mice. Our research indicates that LDLR is a CCHFV entrance receptor and that targeting LDLR pharmacologically may provide a means of treating and preventing CCHFV [42]. Researchers have shown in another study that the LDLR is an essential receptor for CCHFV cell entrance and is crucial for CCHFV infection in blood vessel organoids and cell cultures. Since other members of the LDLR protein family cannot attach to or neutralize the virus, the interaction between CCHFV and LDLR is highly selective. Biosensor assays show that LDLR selectively binds the GPs on the surface of CCHFV. Interestingly, LDLR-deficient mice show a delay in CCHFV-induced illness. Additionally, investigators discovered that CCHFV particles included Apolipoprotein E (ApoE). Researchers demonstrated the critical role that LDLR plays in CCHFV infection, regardless of the presence or absence of ApoE, when the virus is generated in tick cells. This finding has significant ramifications for the creation of CCHFV treatment in the future [43]. The genomic RNPs are released into the cytosol after cell entrance and fusion, and the encapsidated vRNA acts as a template for the L protein to synthesize viral mRNA. The 3′ termini of nairovirus mRNA or the components that stop transcription have not been discussed in any research. On the other hand, the similar nairovirus Dugbe virus has a 7-methylguanylate (m7G) cap containing sequences acquired from cellular mRNA in its 5′ terminal sections. The L protein employs m7G capped primers, taken from cellular mRNA by an endonuclease domain in the protein, to start the synthesis of viral mRNA. As has been shown for endonucleases of other L proteins, the CCHFV L protein has a residue (D693) that is expected to coordinate an Mn2 + essential for the cap snatching action. L protein transcription activity is specifically eliminated by mutation D693, although it retains its capacity to replicate analogs of the CCHFV genome. This implies that CCHFV replication is not initiated by capped primers [13, 14]. Positive sense complementary RNA (cRNA) and uncapped, negative sense vRNA must be replicated and encapsidated for genomic RNPs to replicate. The L protein and NP are very somewhat necessary for the replication of the RNPs. The terms “genomic” and “antigenomic” refer to the encapsidated versions of the vRNA and cRNA, respectively. Using genomic RNPs as a template, capped mRNA is synthesized and antigenomic RNPs are produced since CCHFV is a negative strand RNA virus. To create antigenomic RNPs, NP subunits are added to the elongating strands during the replication of genomic RNPs. The cRNA of the antigenomic RNPs is then utilized as a template to create genomic RNPs. This process is carried out by the L protein [13, 16].

**Fig. 2:**
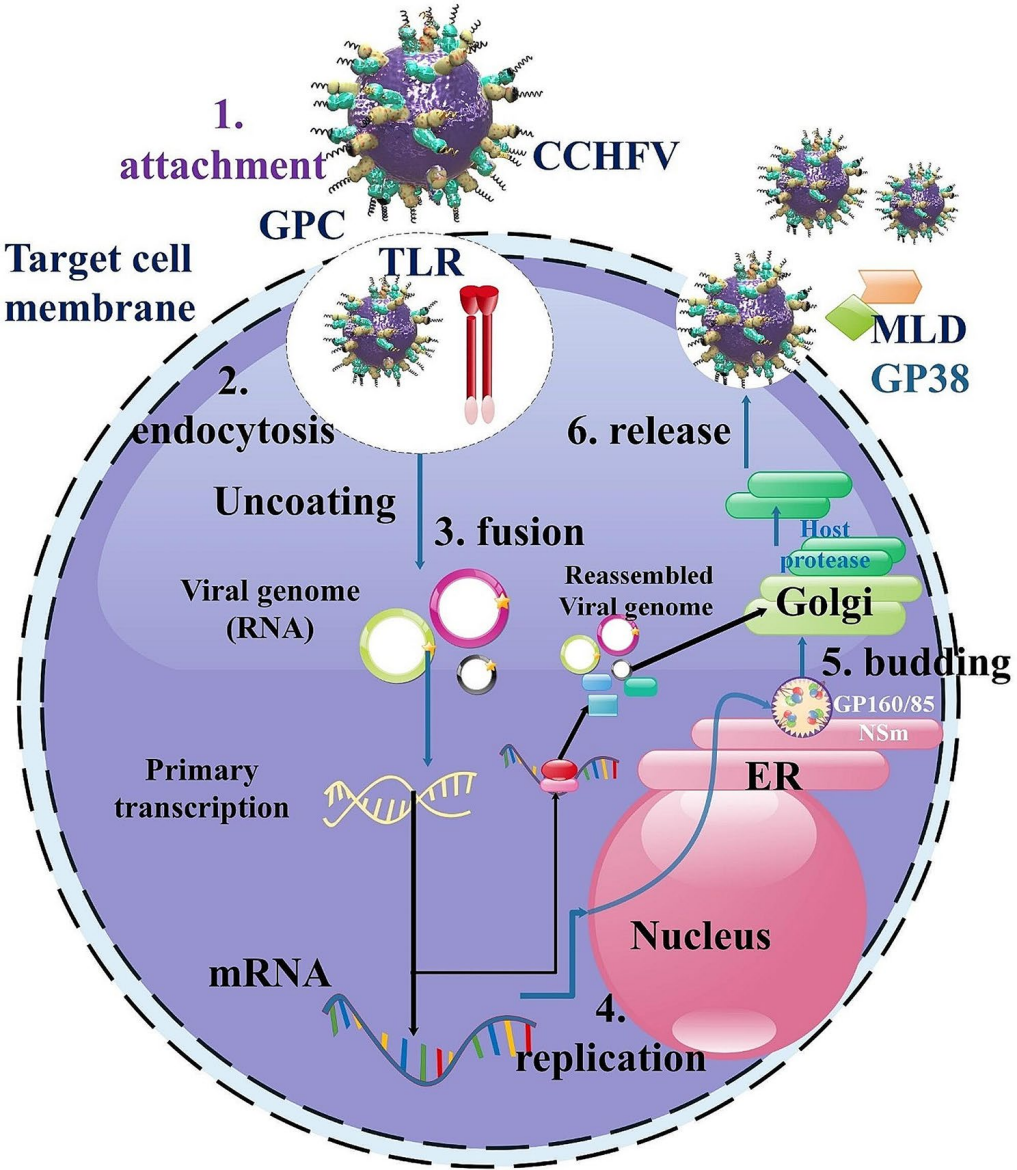
During the first step of attachment, the Crimean-Congo Hemorrhagic Fever Virus (CCHFV) enters the host cytoplasm by relying on clathrin-dependent and pH-dependent mechanisms, which occur in stages 2 and 3, respectively. Upon entering the cytoplasm, viral genomes convert into positive-sense mRNA via the action of the RdRP enzyme. This mRNA then triggers the translation process, leading to the synthesis of viral proteins. Furthermore, these proteins collaborate to generate fresh negative-sense viral genomes enveloped with NP and a bound L protein to commence replication upon infecting the subsequent cell (stage 4). The GPC is transported into the endoplasmic reticulum (ER) and undergoes proteolytic processing through the ER and Golgi apparatus. This processing results in the production of mature GP, as well as the accessory proteins MLD, NSm, and GP38. Recently generated genomes are enclosed inside enveloped particles and the virus emerges from the Golgi apparatus to be released via the secretory route (stage 5). Subsequently, newly formed viral particles are discharged to invade other cells, while GP160/85, MLD, and GP38 are also discharged outside the cells, although the outcome of this release remains uncertain (stage 6). CCHFV proteins not only promote viral multiplication but also inhibit host apoptosis and innate immune mechanisms. The CCHFV NP can impede the intrinsic process of apoptosis at a stage that has not yet. In contrast, the CCHFV NSs facilitate apoptosis by disrupting the mitochondrial membrane or the extrinsic apoptotic pathways. CCHFV may induce apoptosis by generating tumor necrosis factor (TNF) and activating the TNF death receptor pathway. The CCHFV NP is likewise cleaved by host caspase 3, however the presence of oligomeric conformations may hinder this cleavage process. The OTU domain of the CCHFV L protein inhibits the start of the type I interferon response by its deubiquitylating action, suppressing the RIG-I-dependent pathway. MAVS refers to the mitochondrial antiviral signaling protein, whereas vRNA stands for viral RNA [2]

We don’t know anything about CCHFV assembly and egress. Similar to other bunyaviruses, NP is located in the perinuclear area near the Golgi complex, and CCHFV RNPs are most likely present in the cytoplasm. The location of virus budding is frequently determined by the subcellular localization of the viral GPs; accumulation of the GPs in the Golgi complex, trans-Golgi network (TGN), and NP in perinuclear regions is consistent with CCHFV particle assembly and budding in the Golgi complex and/or TGN. Following assembly, polarized epithelial cells exit the basolateral membrane and release the CCHFV particles by exocytosis, often without any visible cytopathology [16] (Fig. 2).

The host often triggers defensive mechanisms during viral infection, including the immune system, cell stress response, and apoptosis for protection. Similarly, after CCHFV infection, several viral or cellular variables have been shown to influence virus-host interactions, affecting the viral infection or host response. The cellular chaperon heat shock protein 70 (HSP70) family and the cytoskeleton component actin may interact with the CCHFV N protein to affect the virus’s ability to replicate. After being challenged by CCHFV, host aquaporin 6, a water channel that facilitates water flow and tiny solutes across membranes, may have a protective function. However, CCHFV may have developed defense mechanisms against specific physiological reactions. As an example, CCHFV may impede the activation of IFN-regulatory factor 3 (IRF-3) and hence delay the type I interferon (IFN-I) pathway. Furthermore, the CCHFV RdRp that has the OTU domain exhibited deconjugating activity against ISGylated and ubiquitinated proteins, hence impeding the innate immunity pathway that is reliant on ISG15 and Ub. Furthermore, it has been shown that the NS protein of CCHFV may cause apoptosis via the death receptor route and the mitochondrial death pathway. Despite these findings, further research is needed to fully understand the host cell response’s worldwide characteristics after viral infection [15, 44–46].

### **Genetic Diversity Crimean-Congo Hemorrhagic Fever Virus**

The viral genome is composed of three separate single-stranded RNA segments: the S segment (1,670 bp), the M segment (5,360 bp), and the L segment (12,160 bp) [47]. CCHFV’s widespread geographical presence is mirrored by a high degree of genetic diversity within the virus population. Although there is substantial conservation across CCHFV strains in the NP and L proteins (95% or more amino acid identity), there is substantially less conservation among CCHFV GPC strains (75% or less amino acid identity). CCHFV clades are separated by physical distance, and CCHFV diversity is positively correlated with location [12, 48]. Phylogenetic studies relying on the S segment have established 6 of 7 CCHFV lineages, each with a distinct distribution [31]. Arboviruses have a high degree of genetic variation, and CCHFV is no exception. Based on the genomic segment under consideration, numerous genotypes are distinguished, and they exhibit geographic segregation by the virus’s origin. CCHFV exhibits a broad distribution spanning thirty countries encompassing Asia, Africa, Europe, and the Middle East. CCHFV is categorized into seven genetic groups according to phylogenetic analysis: two Asian, two European, and three African, which correspond to its geographic origin. In addition, the phylogenetic analysis of the S segment described seven lineages named Africa 1, Africa 2, Africa 3, Asia 1, Asia 2, Europe 1, and Europe 2, while nine and six additional genetic lineages can be characterized for the M and L segments respectively, with however a certain congruent level. Since this happens often with CCHFV owing to segment reassortment, it is noteworthy to note that the phylogenetic tree topology for the same isolate may alter depending on which of the three genomic segments is studied [49–51]. CCHFV is characterized by segment reassortment and recombination, which occur when vectors are simultaneously infected with viral strains from several lineages and produce novel genetic variations [47]. Instead, the cause of genetic diversity may be long-distance migration. The CCHFV strains from the southwest of Europe resemble African isolates more than those from the east, indicating that the virus may have traveled from Africa to Europe by migratory birds carrying infected ticks. The segmented genome of the CCHFV is also capable of reassortment, and isolates of the virus with genomic segments from different geographical lineages have been used to demonstrate historical co-circulation and migration of the CCHFV across great distances [2].

### Host Gene Expression Response

It is unknown what molecular processes occur at the tick-pathogen contact. The contact between the ticks’ epithelial cells and the GPs in the CCHFV membrane is most likely the first stage. It was shown that the glycoprotein Gc was a class II viral fusion protein. Ticks, like other invertebrates, lack adaptive immunity and instead depend on an innate immune response that includes humoral factor release in the hemolymph, phagocytosis, encapsulation, and nodulation. RNA interference (RNAi), a further significant mechanism of arthropods’ (including ticks’) innate antiviral defense against arboviruses, was studied on the Hazara nairovirus, which is regarded as a stand-in for the CCHFV virus. It has been shown that Hazara nairovirus N protein mRNA is the target of small interfering RNAs (siRNAs), which can block virus reproduction. The antiviral action of siRNAs is enhanced when they are coupled with ribavirin. It is unclear exactly what role RNAi plays in tick-CCHFV interactions [52].

The cytoplasmic RNA sensor retinoic acid-inducible gene I (RIG-I) and its adaptor molecule mitochondrial antiviral signaling (MAVS) protein detect CCHFV in cell culture. Proinflammatory and IFN-I responses are both triggered by MAVS. Researchers looked at the function of MAVS in mouse CCHFV infection both with and without IFN-I activity. When IFN-I signaling was active, MAVS-deficient mice were resistant to CCHFV infection and did not exhibit any symptoms of illness. MAVS-deficient mice lost a lot of weight when IFN-I signaling was stopped by an Ab, but they were consistently shielded from fatal illness, whereas all control mice died from infection. The infected MAVS-deficient animals showed a significant blunting of cytokine activity. Further research revealed that CCHFV-infected mice with TNF-α receptor signaling deficiency (TNFA-R-deficient) but not IL-6 or IL-1 activity exhibited more restricted liver damage and mainly were shielded from fatal consequences. In a post-virus exposure scenario, animals treated with an anti-TNF-α neutralizing Ab also showed some protection. Furthermore, investigators discovered that, compared to a deadly strain of CCHFV in mice, a disease-causing but non-lethal variant of the virus caused more muted inflammatory cytokine responses [53].

We still don’t fully understand how the virus affects the host’s innate immune system. The goal of Kozak et al. was to sequence the virus’s genome and evaluate the host immune response in liver cells using RNA-seq, a sort of next-generation sequencing technique. The findings demonstrated the feasibility of concurrently studying viral identification and evolution from the same sample by identifying many changed genes and pathways. Notably, dysregulation was shown for both OAS2 and the genes encoding DDX60, a cytosolic component of the RIG-I signaling pathway. Remarkably, Huh7 cells were able to activate PTPRR but not HepG2 cells. This has been linked to the TLR9 signaling cascade, and TLR9 polymorphisms have been linked to worse patient outcomes. Furthermore, researchers sequenced the whole genome of CCHFV to evaluate the evolution of viral diversity and its correlation with the host response. Consequently, researchers have shown that next-generation mRNA deep-sequencing allows one to analyze viral quasispecies and strain types in addition to mRNA gene expression. This indicates that it is possible to identify the virus and host biomarkers from CCHFV specimens via analysis, which may have consequences for prognosis. This work demonstrates the technique’s potential for characterizing the virus and identifying host biomarkers that may serve as possible indicators of patient prognosis. Furthermore, it could provide crucial pathogenesis-related information before researching animal infection [54].

Researchers have shown that elevated levels of TNF-α and IL-6 play a significant role in bringing about this clinical picture. There are several cytokines in the body that work to counteract this rise. Three members of the TNF superfamily, Fas ligand (FasL), LIGHT, and TNF-like molecule 1 A (TL1A), are bound to the soluble trap receptor decoy receptor-3 (DcR3). Patients with severe CCHF were more likely to have symptoms including fever, bleeding, nausea, headache, diarrhea, and hypoxia than those with mild/moderate CCHF. In CCHF, which has limited therapeutic choices, elevated DcR3 seen early in the illness may enable the testing of new immunomodulatory treatments in addition to antiviral medication [55].

To understand the complex host-viral response, multi-omics system biology approaches, such as biological network analysis, are used to study viral pathogenesis. The goal of researchers was to determine the cellular immune responses that occur during the effective replication of CCHFV in vitro as well as the system-level alterations that follow an acute infection. A system-wide network-based system biology technique was used to assess peripheral blood mononuclear cells (PBMCs) from a longitudinal cohort of patients with CCHF both during the acute phase of infection and one year later during the convalescent phase. An untargeted quantitative proteomics study of the Huh7 and SW13 cells with the highest level of tolerance to CCHFV infection was then conducted. In the RNAseq analysis of the PBMCs, researchers compared the acute and convalescent phases and found that there was a system-level host metabolic reprogramming towards central carbon and energy metabolism (CCEM) with the particular augmentation of oxidative phosphorylation (OXPHOS) after CCHFV infection. Using network-based system biology techniques, it was possible to examine the negative coordination of metabolic pathways during CCHFV infection with biological signaling systems such as the Akt/mTOR/HIF-1 signaling and the FOXO/Notch axis. In Huh7, the cross-sectional proteomics in SW13 cells was consistent with the temporal quantitative proteomics, demonstrating a dynamic shift in the CCEM across time. The two main CCEM pathways—glycolysis and glutaminolysis—were blocked to prevent viral proliferation in culture. The relevance of IFN-I and II-mediated antiviral mechanisms at the systemic level and throughout progressive replication was indicated by the activation of important IFN-boosting genes during infection [56].

## Clinical Presentation

Although the disease’s clinical spectrum ranges from mild to moderate to severe illness, some instances are fatal. Clinical characteristics and non-specific symptoms include weariness, generalized muscle/joint pain, headache, nausea, vomiting, diarrhea, and fever are the first signs and symptoms of CCHF. On the other hand, hepatosplenomegaly, vascular problems, and bleeding occur in individuals with a severe course. The key results in the laboratory include leukopenia, increased liver enzyme levels, thrombocytopenia, and a prolonging of hemorrhagic indicators [12, 57]. There has been a 20–30% death rate in case reports, most likely because practitioners are more prone to publish accounts of patients with severe or terminal diseases. On the other hand, the case fatality rate is often lower for big case series, most likely due to the inclusion of individuals with milder illness; for the over 6000 cases reported from Turkey, it has been 5% [12]. The purpose of a meta-analysis review was to emphasize and provide a comprehensive overview of the CCHF mentioned above characteristics. Africa has a lower mean CCHF mortality rate (22.0%) than both Asia (33.5%) and Europe (33.8%). The highest CCHF mortality rates were seen in the following occupations: agriculture (28.9%), health care (19.2%), slaughterhouse (16.7%), and farmers (13.9%). Numerous clades and genotypes are claimed to be distributed across Africa, Asia, and Europe based on a study of the literature on CCHFV S-segment characteristics. Future research on the epidemiological traits of CCHFV clades, genotypes, and their distribution has an extensive scope [58].

**Fig. 3:**
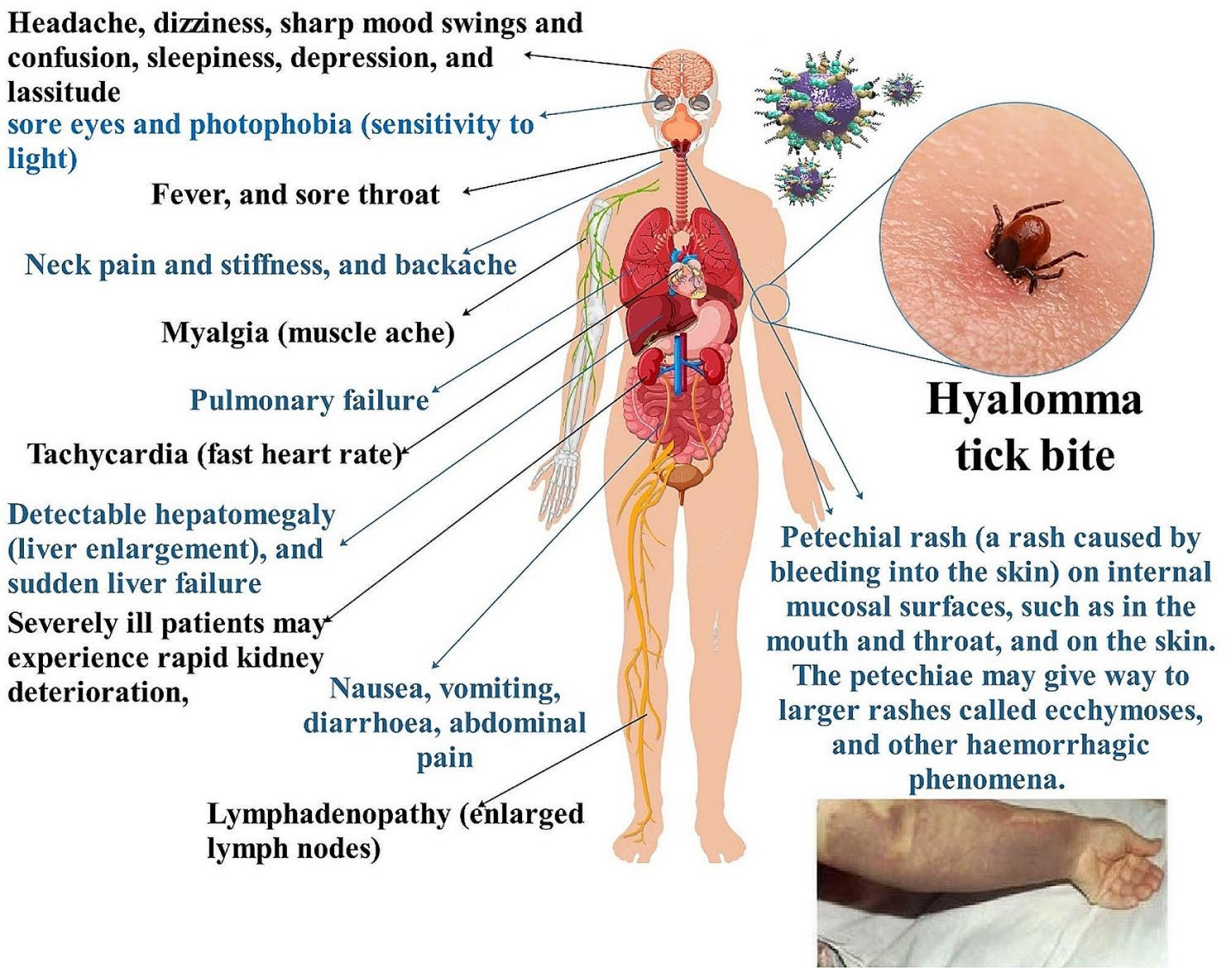
Symptoms of CCHFV The duration of the incubation time is determined by the manner of viral acquisition. The incubation time after a tick bite usually is one to three days, with a maximum of nine days. After contact with contaminated blood or tissues, the incubation period is generally five to six days, with a known maximum of 13 days

The four unique phases of CCHF presentation are incubation, pre-hemorrhagic, hemorrhagic, and convalescence. The incubation time, which varies from 1 to 9 days and depends on the viral dosage and exposure route, is often less than a week. It seems to be shorter after a tick bite (typically 1–3 days) and a little bit longer after coming into contact with the blood, tissue, and secretions of people and cattle that are infected (5–7 days). Pre-hemorrhagic stage symptoms include temperature (39–41 °C), headache, myalgia, dizziness, neck pain and stiffness, backache, headache, painful eyes, and photophobia. It lasts an average of 2–4 days (with a range of 1–3 days). A sore throat, stomach ache, nausea, vomiting, and diarrhea might accompany this. Jaundice, congested sclera, conjunctivitis, and hyperemia of the face, neck, and chest may also be observed64. There have been reports of severe instances causing changes in mood and sensory perception. Anger may give way to calm. There may also be splenomegaly and hepatomegaly. (Fig. 3) [17].

The hemorrhagic phase may last up to two weeks, but it generally lasts just two or three days. Petechia to prolonged ecchymoses on mucous membranes and skin are examples of hemorrhagic symptoms; this finding is more prominent in cases of CCHF than in other viral hemorrhagic fevers (VHFs). Bleeding from injection sites is frequent, as are hemoptysis, melena, hematuria, epistaxis, and hematemesis. There have been isolated reports of bleeding from the brain, uterus, and vagina. In addition to increased levels of inflammatory cytokines, hematology, and blood chemistry often reveal thrombocytopenia, leukopenia, and raised levels of aspartate aminotransferase (AST), alanine aminotransferase (ALT), lactate dehydrogenase (LDH), and creatine phosphokinase. Prolonged prothrombin and activated partial thromboplastin durations may impact coagulation, causing a drop in fibrinogen levels and an increase in fibrinogen breakdown products. In severe instances, the haemorrhagic stage is prominent and rapidly progresses to shock, disseminated intravascular coagulation (DIC), overt bleeding, and renal, hepatic, or pulmonary failure. If fatal, most deaths happen within the second week of the sickness [59–61].

Convalescence in survivors usually starts 9–10 days (range 9–20 days) after the disease begins and is characterized by a return to normal laboratory results. This period may last longer than expected and is linked to several conditions, including memory loss, hair loss, polyneuritis, breathing problems, xerostomia, tachycardia or bradycardia, hypotension, and visual and hearing impairments. Relapse and a biphasic course of the illness are not supported by credible data; conversely, the sequelae have not been well explored to identify long-term consequences. Generally, survivors acquire cellular and humoral immunity to CCHFV [17, 62, 63] (Fig. 3).

For instance, CCHF was identified at a Senegalese hospital for the fourth patient—a foreign national—in July 2023. The patient was a merchant in his 50s who lived in the capital of a nation that borders Senegal. It’s possible that he had close encounters with animals at home or work. Two days after arriving back at his remote home on July 16, 2023, he started experiencing fever, headaches, and stomach discomfort. His symptoms prompted a checkup at a home country private hospital, where a course of therapy was started the patient did not get well. The patient’s condition worsened two days after seeking evaluation at a referral hospital in his own country due to ongoing clinical complaints. His abdominal ultrasonography showed hepatopathy in addition to petechiae, and the family decided to go to Senegal for better medical treatment. Based on biological tests and fibroscopy performed in Senegal, the findings indicated severe thrombocytopenia with 2,000 platelets/µL (reference range: 150,000–450,000/µL). The patient was subsequently taken to the critical care unit of the National Hospital of Pikine, Dakar, due to a deteriorating clinical state that included hyperglycemia and hematemesis. Blood samples were taken for biologic examination by the health district team on the third day of the patient’s stay in an intensive care unit. Ten days after the disease started, PCR testing revealed a positive result for CCHFV; however, the patient passed away from multiorgan failure on the same day the PCR results were received. The research team found 38 connections in the patient’s nation of origin throughout the case study; 46 contacts were found in Senegal, with the majority (87.7%) being medical professionals, such as physicians, nurses, and laboratory technicians. There were no documented bloodborne pathogen exposure while providing patient care or managing the patient’s samples. On the other hand, there wasn’t much infection prevention and control (IPC) going on. Using a standardized evaluation, researchers evaluated the IPC level by asking questions about waste management, material sterilization, personal protective equipment use and availability, and IPC training for healthcare staff. Despite the relatively low IPC level, investigators managed this CCHF case and discovered no recorded occurrences of blood exposure. After the 14-day contact follow-up, no additional CCHF cases were detected [64].

## Standard Molecular Detection

**Fig. 4:**
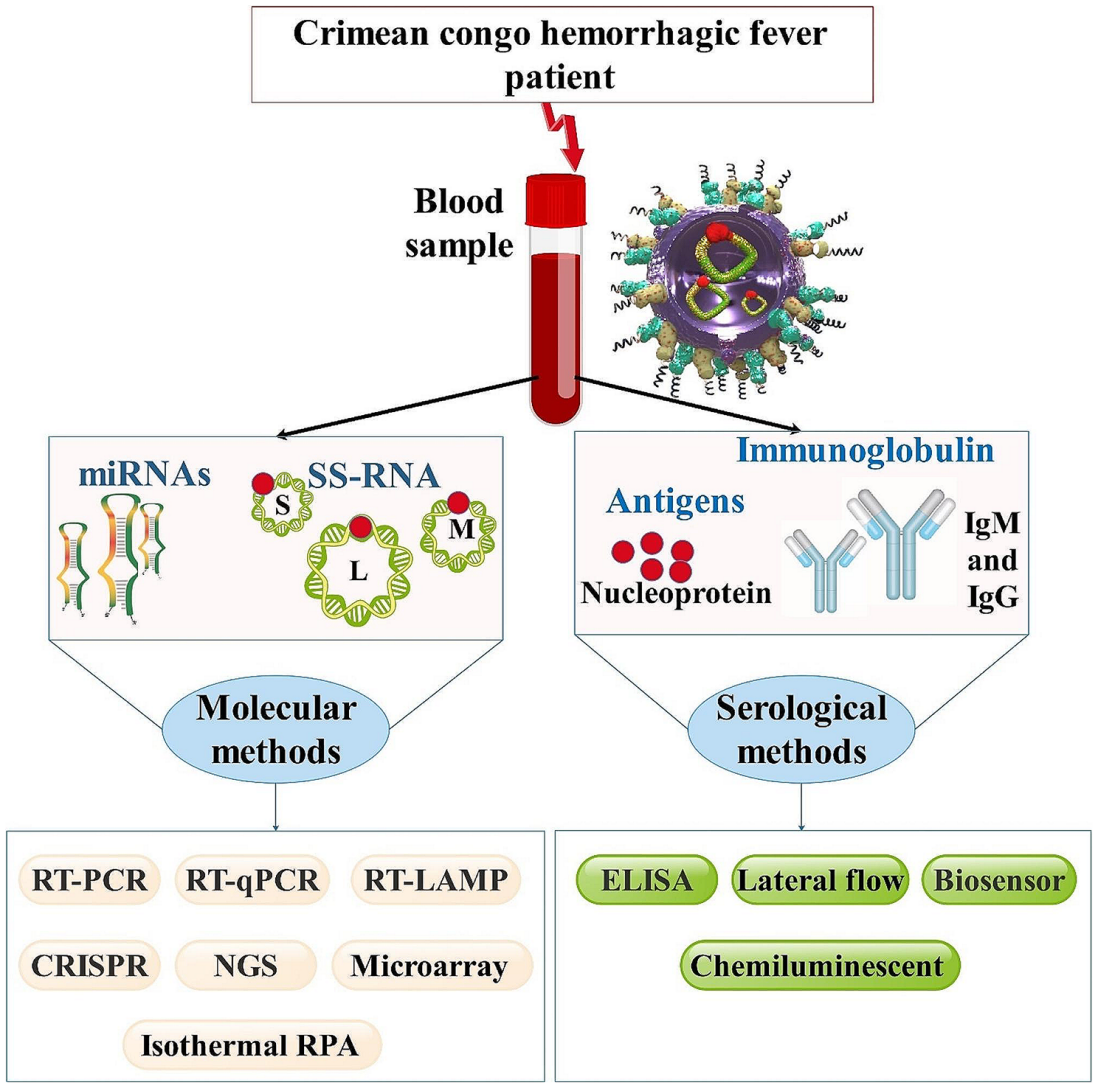
A diagram depicting the various analytical approaches available for CCHFV detection. Blood samples are taken to identify viral NA and human Ab to the virus. CCHFV can be diagnosed using a variety of molecular and serological testing

Generally, there are two types of CCHFV diagnostic methods: direct and indirect tests. Direct techniques, which are advantageous in the early stages of the disease, involve isolating the virus via cell culture, diagnosing the viral antigen, and detecting the virus genome. Serological techniques, including direct immunofluorescence (DIF), passive hemagglutination assay (PHA), complement fixation assay, and ELISA, are examples of indirect approaches for detecting antibodies (Ab) versus the virus [60, 65, 66]. Anti-CCHFV IgM Ab may be found anywhere from one week after the start of the illness to four months following infection. IgG responses quickly follow IgM Ab throughout CCHF infection. IgG Ab against CCHV is detectable for at least five years. By detecting anti-CCHFV IgM Ab or specific IgM and IgG Ab simultaneously in an initial serum sample, an acute phase CCHFV infection may be serologically validated. Seroconversion or at least a four-fold rise in IgM Ab titer in matched serum samples may be used to confirm recent or ongoing infection. Elevated specific IgM levels indicate a recent infection, but they do not establish CCHFV as the origin of the symptoms. Seropositivity in regions where CCHFV is prevalent may indicate a recent, mildly symptomatic infection. Other illnesses including dengue, malaria, or Q fever may elicit similar symptoms. As a result, the diagnostic utility of seropositivity is contingent upon the local VHF prevalence. Positive IgM findings should be considered together with the patient’s recent travel history in areas where CCHF is not prevalent. Anti-CCHFV IgG Ab by themselves are unable to establish a continuous CCHFV infection. Since an Ab response is seldom seen in instances that end in death, it is a sign that the condition will not be deadly [67]. The clinical symptoms of CCHFV are similar to those of many other illnesses, making diagnosis difficult. For this reason, it highlights the need to develop new biomarkers to help diagnose CCHFV. Platelets, AST, ALT, first-step coagulation assays, LDH creatinine, and fibrinogen have all been identified as laboratory indications of CCHFV [68–70]. After the acute phase of the infection, which lasts 4–9 days after symptoms appear, active CCHFV infection may be identified by IgM or a substantial rise in IgG titer; however, severe and fatal cases often do not develop a detectable Ab response. Anti-CCHFV IgG may be found in the blood and helps monitor epidemiological investigations. It can indicate ongoing or resolved infection (many years after infection). It has been shown that capture ELISA is more sensitive for CCHFV than either IFA or neutralization test. Since CCHFV elicits comparatively modest levels of neutralizing Ab, virus neutralization tests are less effective for diagnosis, but they may be helpful for epidemiology and vaccine development. Plaque reduction neutralization is often used for CCHFV neutralization, and effects take 5–7 days to manifest [71]. Solid-phase radioimmunoassay technique (SPRIA), real-time PCR (RT-PCR), and viral isolation (tissue culture or using a mouse model) are some more laboratory techniques. ELISA and SPRIA are the most recent sensitive, fast, and repeatable procedures for detecting viral antigens and Ab in a short period (5–6 h) [72, 73]. Nonspecific symptoms, genetic variety of CCHFV, and biosafety requirements for managing a virus of high biological risk all contribute to the difficulty of diagnosing CCHF. There are several laboratory tests available; selecting one relies on the patient’s symptoms, the accessibility of samples, and the reliability of the test for the CCHFV strains prevalent in the area where the infection was likely acquired. After the first week of sickness, serological approaches have the best chance of being effective since they are less affected by tiny genetic differences. However, serological tests are likely to provide false-negative findings during the early acute phase of the illness, making molecular approaches the method of choice [74] (Fig. 4) (Table 1).

**Table 1:** Nucleic acid amplification tests for CCHFV

Method type	Genomic Target (segment)	Number of CCHFV strains and/or patient samples tested	Limit of detection (LOD)	Other viral hemorrhagic fever viruses	REF
RT-PCR	S	Kosovo	2,779 geq/ml	MBGV/EBOV, LASV, RVFV, DENV, YFV	[75]
RT-PCR	S	18 strains	5 RNA copies/reaction	-	[76]
Isothermal recombinase polymerase amplification	S	12 strains/ human and tick samples from Tajikistan	50–500 copies	-	[77]
Multiplex amplification followed by next-generation sequencing	L	Turkey	-	YFV, RVF, EBOV, JUNV, CHIKV	[78]
RT-PCR	S	Southern Africa	-	-	[79]
Multiplex RT-PCR combined with universal array	S	3 strains	190 RNA copies/ml	EBOV, MBGV, LFV, YFV, DENV	[80]
One-step RT-PCR	S	Kosovo	30 PFU/ml	-	[81]
RT-PCR	L	1 strain	33–100 fg/µl	EBOV, MBGV, LFV, GTOV, BHF, JUNV, BzHF, SEOV, PUUV, HPS, RVF	[82]
RT-PCR combined with a DNA microarray	L	4 strains/ the Balkans and the Middle East	10^5^–10^6^ PFU/ml amplified cDNA	LASV, BHF, GTOV, HPS, PUUV, SEOV, DOBV, SNV, RVF, KFD, YFV, TBE, RESTV, EHF, MARV, NiV, HeV,	[83]
RT-PCR	S	18 strains; patient samples from Uzbekistan	11.8 copies	-	[84]
RT-PCR	S	1 strain	5 PFU	-	[85]
RT-PCR	S	8 strains; patient samples from Turkey	11 genomes/reaction	-	[86]
RT-PCR	S	Animal and tick samples from India	7.6 copies	-	[87]
RT-PCR	S	16 strains	256 PFU/ml	-	[88]
RT-LAMPa	S	Patient samples from Sudan	10 fg (naked eye turbidity), 0.1 fg (agarose gel electrophoresis)	-	[89]
RT-PCR	S	Patient samples from Albania	-	-	[90]
One-step real-time RT-PCR	S	4 strains	2 copies/lXXX	-	[91]
RT-PCR	S	Patient samples from the United Arab Emirates	-	-	[92]
One-step RT-PCR combined with a DNA macroarray	S	18 strains; patient samples from Iran, Namibia, Pakistan, and South Africa	6.3 genome copies/reaction	-	[93]
RT-PCR	S	12 strains; patient samples from Iran, Pakistan, and South Africa	1,164 copies/ml	-	[94]
One-step RT-PCR	S	Patient samples from East Anatolia	102 genome equivalents/ml	-	[95]
One-step RT-PCR	S	Patient samples from Iran	20 RNA copies/reaction	-	[96]

### Standard Reverse-Transcriptase PCR (RT-PCR)

Molecular techniques including PCR, one-step RT-PCR, and Nested RT-PCR are sensitive, fast, and precise methods that are perfect for the early diagnosis of individuals suspected of having CCHFV infection [97]. Multiplex nested PCR (DNA viruses) or multiplex nested RT-PCR (RNA viruses) is a variation of PCR in which two or more loci are amplified simultaneously in a single reaction, thereby improving the reliability of detection [98, 99]. To identify CCHFV, traditional RT-PCR assays were created and assessed. However, in order to boost sensitivity and verify the identification of the initial amplified PCR product, the majority of traditional RT-PCR tests include a second round of nested amplification or nucleic acid hybridization experiments. It is well known that since nested PCR involves several alterations of the original PCR results, it is error-prone, time-consuming, and complex owing to cross-contamination. In order to quickly identify CCHFV targeting the short (S) RNA segment, a reverse transcription (RT) loop-mediated isothermal amplification (RT-LAMP) test was created and compared to nested RT-PCR in the current work. The S segment of the viral genome’s highly conserved section served as the template for a set of RT-LAMP primers that were used to identify every strain of Sudanese CCHFV. CCHFV strains may be easily and quickly detected using RT-LAMP. A 10-fold serial dilution of CCHFV RNA was tested, and the findings of the RT-LAMP and the nested RT-PCR agreed 100% of the time. Due to the repeated modification of the main PCR products, nested PCR is complicated by cross-contamination and error-prone. High levels of diagnostic sensitivity and specificity are achieved by the RT-LAMP when testing different acute-phase sera that are obtained during CCHF epidemics. Because the RT-LAMP can be performed in an isothermal environment without the need for specialized equipment and results can be seen with the unaided eye, it is a more cost-effective and practical assay than nested RT-PCR in low-resource economies [100]. In a study, Kalvatchev et al. present a case of this VHF in a 66-year-old man from Sliven that was confirmed by the above-mentioned molecular methods. Modified and adapted (by the authors) one-step Real-time RT-PCR and Nested RT-PCR to demonstrate the virus of CCHF are fast, specific, and sensitive methods suitable for early diagnosis of patients suspected to have this infection [97]. In addition, the RT-PCR approach was employed to identify the CCHFV genome in ticks based on the S segment. Investigated the prevalence of CCHFV virus infection in hard ticks from Iran’s South-Khorasan province. 684 sheep, goats, cows, and camels were sampled in this study from the counties of Birjand, Qaen, Khusf, Darmian, and Sarbisheh. Tick genus and species were identified using a stereomicroscope and valid morphological keys. The CCHFV genome was found in 7 out of 100 ticks using RT-PCR. Ticks that tested positive belonged to the *Hyalomma* and Rhipicephalus genera. All infected ticks were recovered from goats and sheep in Birjand [101]. The findings of this work show a complete reference for future research on tick systematics, population genetics, molecular epidemiology, and tick evolution [102].

Real-time quantitative reverse transcription PCR (RT-qPCR) has proven more influential for CCHFV detection and investigation over the last decade. It provides a reliable, quick, and risk-free method for identifying the biosecurity level 4 pathogen CCHFV in laboratory equipment with fewer biosecurity requirements [91]. The considerable genetic diversity of CCHFV has always been a major challenge in developing RT-qPCR for this virus [103]. Using degenerate nucleotides in the primers/probe is the most popular strategy for covering all CCHFV variations. Although adding primers is beneficial, more probes may need special attention when working with a particular quencher. Using an excessive number of probes may act as a powerful quencher and reduce the sensitivity of the experiment. To cover all CCHFV genotypes, the most typical technique is to insert extra primers and probes that target particularly varied CCHFV strains to cover all genotypes of CCHFV. Another possibility is to direct the forward primer to the S-5’ segment’s non-coding *Orthonairovirus*-specific end. In contrast, Miriam A. Sas and colleagues created distinct primer sets for each CCHFV genotype. In addition, one degenerate primer pair and two probes were introduced to identify all genotypes. 6 genotype-particular synthetic RNAs and associated deactivated virus strains, as samples from numerous animal species, humans, and other Bunyavirales members, were used to test and evaluate the innovative RT-qPCR method. The synthetic RNAs utilized in this RT-qPCR make it possible to separate accurate positive outcomes from undesirable laboratory contaminants. The RNAs that have been generated can also be used as calibrators for genotype-specific measurements [91].

### Loop-Mediated Isothermal Amplification

From the early 1990s, the isothermal amplification of nucleic acids assays has appeared as a critical detection technique, with uses in clinical detection. Loop-mediated isothermal amplification (LAMP), nucleic acid sequence-based amplification (NASBA), helicase-dependent amplification (HDA), strand displacement amplification (SDA), rolling circle amplification (RCA), recombinase polymerase amplification (RPA), transcription-mediated amplification (TMA) were among the techniques used [104]. Isothermal amplification (IA) of nucleic acids (NA) reduced the requirement for temperature cycles, increased enzyme action, decreased sample use, and reduced time to provide quick outcomes. Similarly, a completely closed and secure micro-structured machine can decrease the potential for pollution [105]. IA-based assays are more accessible, easy, ordinary, conscious, transportable and need less energy than PCR/RT-PCR. They can also meet the demand for an easy, prompt, accessible, and molecular detection technique without culture for universal CCHFV assessment [106, 107]. This method employs 3 enzymes: recombinase, single-strand binding proteins (SSBs), and strand-displacing polymerases. By first partnering with the primer, the recombinase enzyme connects to the target sequence. To stabilize the DNA, the SSBs link to the displacement DNA strand and create a “D” loop-like construction. This displaced DNA has a free 3′-hydroxyl region where DNA polymerase may connect and augment the target sequence. As a result, within 20 min, both strands are multiplied exponentially. To visualize the amplified output, fluorescent or non-fluorescent probes can be utilized. RPA is simply multiplexed via different primer sets and may identify multiple infections concurrently in minutes [71, 108, 109]. LAMP, a revolutionary DNA amplification technique, has been used to diagnose infection. Using reverse transcriptase, RNA sequences can be amplified using reverse transcription LAMP (RT-LAMP). RT-LAMP approaches can identify viruses, including Rift Valley fever virus (RVFV) and CCHFV [110]. Jyoti S.Kumar and colleagues used a fast and susceptible one-tube RT-LAMP test to diagnose CCHFV. The CCHFV-particular RT-LAMP test was also validated using human and tick samples. The test identified multiple CCHFV isolated, suggesting that it applies to a wide range of strains. When the RT-LAMP test was compared to RT-PCR, there was complete coordination, as well as 100% sensitivity and specificity. There was also no interaction with related Flaviviruses or HFV discovered. The test is a basic molecular diagnostic that may be implemented on a portable thermal block device [111]. To compare the outcomes of RT-qPCR and colorimetric RT-LAMP tests, researchers at the University Hospital of Salamanca were able to collect plasma and urine samples from patient P4 on days 1 through 13 of his hospital stay. In contrast to the RT-LAMP test, which remained positive until day 8, the RT-qPCR assay in plasma samples was positive on days 1 and 3. According to some publications, blood samples may be used to identify CCHFV RNA by RT-qPCR for up to 18 days from the onset of the illness, with the first five days showing the best results. It has been seen up to 36 days following the first infection. On the other hand, there is currently no information on the molecular identification of CCHFV RNA in plasma samples during infection. Compared to RT-qPCR, RT-LAMP should be more sensitive for detecting vRNA in plasma samples from CCHFV patients. Urine samples were only positive for CCHFV using RT-qPCR the day following admission; however, RT-LAMP detected the virus eight and thirteen days later. Previous studies have also shown that vRNA survives in urine samples beyond serum clearance and that viral load in urine samples is equivalent to that in blood. RT-LAMP amplified both genotypes and showed better sensitivity in urine samples than RT-qPCR. Thanks to a novel, rapid, precise, and sensitive RT-LAMP test created by researchers, different CCHFV genotypes may now be identified in clinical samples. Using our pan-CCHFV RT-LAMP, vRNA was discovered for the first time in urine samples. Using a simple, affordable, real-time single-tube isothermal colorimetric approach on a portable platform, molecular diagnostics may now be offered to rural or resource-limited areas where CCHF often arises [74]. Replication protein A (RPA) is an isothermal technique for denatured genomic target DNA utilizing recombinase-primer compounds and ss-DNA stabilization using ssDNA binding proteins. The RPA diagnosis method is analogous to Taq-Man hydrolysis probes. The difference is that the probe contains tetrahydrofuran, a basic location analog cleaved via endonuclease IV. Because proteins are active components in DNA denaturation, high denaturation temperatures are unnecessary. As a result, the reaction occurs at temperatures between 37 and 42 °C and is quicker than the PCR procedure, often lasting 5–7 min. Because of RPA’s great sensitivity, it can identify tens of copies of the target [112–114]. An isothermal RPA assay for CCHFV detection has been successfully developed. The test detects viral components or synthetic vRNA from all 7 S-section clades of CCHFV in less than 10 min, with high target specificity [77].

### Standard Immunological and Serological Detection

Enzymes are powerful virus diagnostic tools, including applications such as enzyme immune assays and ELISA. The fluorescence polarization immune method (FPI), micro-particle immune method (MEI), and chemiluminescent immune test are all examples of enzyme immune assay applications (CLIA). ELISA uses antigen-Ab interactions with conjugated tags, such as fluorescent tags, and chemiluminescent tags supplemented with substrates, such as polarized light and fluorescent substrates. CCHFV detection was made sensitive and specific by using particular IgM and IgG Ab in human serum and recombinant CCHFV NP as antigen absorption and IgG immune complex (IC) ELISA testing. These recombinant proteins were produced using various expression methods, and an indirect ELISA was created to identify them [115]. Anti-CCHFV IgM and IgG Ab are accurately detected by available serological test techniques. However, their diagnostic efficacy varies depending on the stage of infection [67]. The CCHFV NP antigen is the most often utilized antigen for the serological tests of CCHFV diseases in humans [23]. There is currently just one commercially available fast test for identifying CCHFV Ab. CORIS BioConcept (Belgium) created the CCHFV Sero K-SeT lateral flow test kit to identify IgM-specific Ab in the patient’s plasma, cf. However, research conducted on patients in Iran by Vahid Baniasadi et al. revealed a relatively low sensitivity of 39.7% and a specificity of 92.9%. There is no commercially available fast test for detecting the CCHFV virus directly. Due to its low sensitivity, the CCHFV Sero K-SeT was shown to be unsuitable for screening CCHFV-suspected patients [116].

Nanobodies generated from derived single-chain Ab have shown to be practical tools in diagnostics, treatments, and research into the structure and function of membrane receptors. The researchers used lymphocytes from an alpaca inoculated with the recombinant mouse Kupffer cell receptor Clec4F, which binds to galactose and N-acetylgalactosamine and is vital in pathogen detection. The researchers created a series of nanobodies that target several diagnosis epitopes of the Kupffer cell-particular receptor Clec4F, which might benefit structural and functional study, molecular imaging, and therapeutic medications. Clec4F comprises a Ca^2+^-dependent (C-type) carbohydrate-recognition domain (CRD) for detecting glycans, an N-terminal cytoplasmic signaling domain, and a transmembrane hydrophobic helix, and a heptad neck zone for trimer organization. Clec4F is detected only in Kupffer cells and not in invading monocytes. Consequently, Clec4F can be used as a particular Kupffer cell marker to investigate the functions of various populations in the liver. The lack of Clec4F + Kupffer cells in mice infected with CCHFV is a marker of significant liver damage caused by CCHFV infections [117] (Table 2).

#### Antigen Detection ELISAs

Because NP is highly conserved and immunogenic, the NP of CCHFV is utilized as an early diagnostic marker. As adsorption Ab and production detectors, particular polyclonal and monoclonal Ab against NP can be used. The researchers provide a speedy and sensitive antigen capture ELISA based on double Ab to detect CCHFV. The method detected viral NP in various media, such as human serum, ticks, and culture supernatant. The LoD of the sELISA test is 25 ng of purified antigen. Its LoD was discovered to have 1000 CCHFV genome equivalents compared toRT-PCR. Furthermore, the test was compared to a marketable kit using gamma-rays CCHFV and found to be 100% sensitive and specific. The recently advanced sELISA, with great sensibility and specificity, might be utilized to detect the CCHFV virus in patients, ticks, and culture supernatants. The test will be beneficial as a substitute technique for detecting acute infections and will allow to screen large samples in source-constrained adjustments [118]. Another study is designing and verifying a new CCHFV double-antigen ELISA for detecting anti-CCHFV NP Ab. Because it is based on recombinant NP of the IbAr10200 virus, the ELISA may be performed in standard biosafety situations. The double-antigen sELISA (DA-sELISA) was demonstrated to be a very selectable, accurate, and particular diagnostic for CCHFV-stimulated Ab. It allows for the analysis of many species simultaneously with an identical procedure. As a result, it combines multiple experiment qualities: excellent sensitivity and specificity, as well as great result commensurability [119].

Furthermore, the study provides a species-independent competitive ELISA (cELISA) for diagnosing CCHFV-particular Ab. For this objective, they developed a nucleocapsid (N) protein-specific monoclonal Ab against *Escherichia coli* (*E. coli*) produced as CCHFV N-protein. The most competitive mAb was used to build the cELISA. The detection sensibility and accuracy of the cELISA were assessed to be 95% and 100%, respectively, with 2% of the serum providing inconsistent findings. This cELISA paves the way for future wide-reaching screening methods in various animal species to assess vulnerability to CCHFV diseases and to detect and screen CCHFV [120] (Table 2).

#### IgM/IgG Detection ELISAs and LFIs

From the sixth day of CCHFV disease, IgG and IgM Ab can be identified in serum using enzyme-linked immunoassay (EIA) or ELISA. IgM or a 4-fold enhancement in the titer of IgG Ab in blood samples between the acute and convalescent phases can aid in disease diagnosis. IgM levels can be detected for up to four months, while IgG levels can be detected for up to five years. Patients with deadly diseases do not frequently have a detectable Ab response, hence viral detection in blood or tissue samples is used to diagnose them, as well as in the initial few days of sickness. Immunofluorescence or EIA can occasionally detect viral antigens in tissue samples [121]. Scholars describe the creation and verification of two innovative ELISAs (BLACKBOX CCHFV IgM, BLACKBOX CCHFV IgG) that utilize recombinant CCHFV NP as an antigen to detect CCHFV-specific IgM and IgG Ab. The performance of the test was assessed using commercially available ELISA kits (VectoCrimean-CHF-IgM/IgG; Vector-Best) and in-house gold standard testing (CCHFV IgM/IgG immunofluorescence test IIFT). The evaluation was conducted using a meticulously characterized serum panel collected from 15 Kosovar patients diagnosed with CCHFV via RT-PCR between 2013 and 2016, including samples from both the acute and convalescent phases of the disease. Both IgM ELISAs, including the CCHFV IgM IIFT, identified CCHFV-specific IgM Ab in all sera obtained on or after day 5 after the onset of symptoms during the acute phase of the disease. However, in detecting the increasing IgG Ab titers in samples obtained between days 11 and 19 after the onset of symptoms, the BLACKBOX CCHFV IgG ELISA and the CCHFV IgG IIFT were significantly more sensitive than the VectoCrimean-CHF-IgG ELISA [122].

In a different study, researchers used samples from healthy blood donors obtained in Kosovo and samples from CCHFV patients to assess the diagnostic capabilities of 10 serological assays based on the detection of IgM and IgG Ab against CCHFV. Since the goals of IgM and IgG testing differ, the studies concentrated on the infection phase. Researchers concluded that although the tests differed in their different diagnostic capabilities according to the infection phase, they are appropriate for accurately detecting anti-CCHFV IgM and IgG Ab. IgM tests work effectively in the early and convalescent stages of illness and are primarily used to help the diagnosis of acute infections. The ELISA test’s sensitivity for identifying specific IgG Ab varied depending on whether the illness was in the early or convalescent stages. The maximum yield was obtained using the BlackBox IgG ELISA, followed by the EUROIMMUN IgG ELISA and the VectorBest IgG ELISA, which had the lowest sensitivities. The VectorBest IgM ELISA found that many samples had anti-CCHFV IgM Ab in the subsiding phase. When detecting anti-CCHFV IgM Ab during both the acute and convalescent stages of infection, the two immunofluorescence-based test techniques had the same sensitivity. IgG tests have the maximum sensitivity during the infection’s subsiding phase, making them pertinent for disease monitoring and later phases of illness development [67].

The conserved recombinant NP was used in this work as a scalable and safe alternative antigen for creating an indirect ELISA detection platform for IgM and IgG. Via the use of suspected clinical samples gathered from India’s hotspot regions, the indirect ELISA was assessed. Relative to reference MAC ELISA and IgG ELISA, there was a 95% and 100% correlation, respectively. The findings show that the developed indirect ELISA for measuring IgM and IgG has good sensitivity and specificity for identifying human CCHFV Ab. These assays are simple and may be used for serosurveillance and high throughput screening of human samples for clinical diagnosis. Additionally, these assays may be converted into affordable point-of-care testing formats for use in resource-constrained environments [123].A study comprised patients at Boo-Ali Hospital in Zahedan, Iran, whose hemorrhagic fever (HF) symptoms ranged from moderate to severe. Every patient had two blood samples drawn: one when the patient met the requirements for CCHF notification and the other two weeks after the patient’s hospital release. Six patients’ acute serum samples had a positive IgM signal, according to in-house and commercial testing. One discovery was that CCHF patients acquire neutralizing Ab quickly after infection. It’s interesting to note that additional CCHF virus strains might also be neutralized by similar Ab. An original clinical sample from a single patient with a proven case of CCHF infection was used to determine the whole sequence of the Zahedan 2007 isolate, including the previously unidentified first L-segment sequence in question [124].

Using all available complete sequence data for the S gene encoding the CCHFV NP and Ab cross-reactivity between the NP of a Greek isolate (AP92), the most genetically diverse CCHFV strain, and the NP of a South African isolate, the other study aimed to determine genetic diversity in CCHFV. A collection of 45 CCHFV isolates taken from GenBank was used to determine the pairwise distances, nucleotide sequence diversity, and amino acid diversity within and across genotypes. When compared to SPU415/85, which was isolated from a human infection in South Africa, the most diversified virus, AP92, which was isolated from a tick in Greece, showed the largest amino-acid change (8·7%). The codon-optimized S genes of SPU415/85 and AP92 encoded recombinant NP, which was produced in a bacterial host system and used to create an in-house ELISA to identify IgG Ab against CCHFV in South African patients who recovered from diagnosis. Thirteen out of fourteen sera responded with the Greek recombinant NP, while 14 reacted with the South African recombinant NP. The two NP antigens’ serological cross-reactivity indicates that recombinant antigens made from geographically different strains of CCHFV will be used for epidemiological and diagnostic purposes globally [125].

The development and assessment of safe, sensitive, and specific IgG indirect ELISA (iELISA) using recombinant NP of the CCHF virus as an antigen is the subject of separate research. The NP gene sequence that had been codon-optimized was created, cloned, and expressed in the pET28a + vector. After affinity chromatography was used to purify the recombinant NP to homogeneity, Western blot and MALDI-TOF/MS analysis were used to characterize it. Using a panel of animal sera, the identified protein was used to create an indirect IgG microplate ELISA. Using 76 suspicious samples, the internal ELISA was compared to a commercially available ELISA kit (Vector-Best, Russia). The results showed a 90% concordance with 79.4% sensitivity and 100% specificity, respectively. The test is reliable and repeatable under various circumstances, according to the precision analysis. Additionally, the test was used for serosurveillance in ruminants from various parts of India, and the results showed that 18% of the ruminants were seropositivity, suggesting that the virus was still in circulation in the area. According to the results, the new IgG iELISA that uses recombinant NP is a valuable and safe method for scaling up high-throughput screening of Ab specific to CCHFV in various species [126] (Table 2).

**Table 2:** ELISA and the serological diagnosis of CCHFV

Method type	Detection Target (segment)	Comparison with reference	Characteristics	REF
Sandwich ELISA (sELISA)	Nucleoprotein (NP)	RT-PCR	High sensitivity and specificity, identification of CCHFV virus in people, ticks, and culture supernatant, large-scale sample screening in resource-constrained settings	[118]
Recombinant ELISA (Rec-ELISA)	Recombinant NP / Mucin-like variable domain (rNP/rMLD)	RT-PCR	High sensitivity and specificity	[126]
Indirect ELISA (iELISA)	(rNP)	In-house iELISA and VectoCrimea CHF IgG ELISA kit	Sensitivity, specificity, repeatable in diverse sets of situations, safe, stable, and scalable	[127]
Competitive ELISA (cELISA)	rNP	Sera from CCHFV-endemic locations was previously described using an adapted commercial ELISA.	Large-scale screening, test species independently, high sensitivity and specificity	[128]
Double-antigen sandwich ELISA (DA- ELISA)	NP	Species-adapted VectorBest ELISA and Euroimmun IFA	Highly sensitive and specific, testing several different species at the same time, minimizing the false-positive	[129]
Immune complex (IC) ELISA and µ-capture ELISA	NP	IgM/IgG indirect immunofluorescence (IIF) testing in-house and commercially accessible IgM/IgG ELISA assays	Sensitivity, specificity, and ease of use are all advantages of producing viral antigens in high quantities in *E. coli* without needing a cost-effective eukaryotic expression system or native virus rearing.	[130]

## Advanced Detection

Based on the vital importance of early identification of CCHF in infection control, the development of a biosensor as a fast tool looks to be highly significant in detecting CCHFV; nevertheless, there have been few investigations on this issue, and further research is required. The CCHFV biosensor platform may be thought of in three stages: (1) diagnosis of CCHFV nucleic acid or proteins, human immunoglobulins, and human miRNA, (2) use of an immobilized bioreceptor on a transducer containing a DNA probe, Ab/antigen, ligand, aptamer, and enzyme, and (3) hybridization detection methods such as electrochemical, piezoelectric, fluorescent, colorimetric, magnetic, and acoustic technologies [131, 132, 133].

### Biosensors

Aptamers are considered a particular and sensitive instrument that may be employed in quick diagnostic approaches [134]. The CCHFV NP was chosen as the target for aptamer separation because of its affluence and conventional construction between other viral proteins. The surface plasmon resonance (SPR) technique was used in one investigation to measure the binding affinities (Kd values) of aptamers. To create the aptamer-Ab ELISA test, the Apt33 with the greatest desire to NP was chosen. It effectively identified CCHFV NP in human serum at a value of 90 ng/ml. The test’s specificity and sensitivity were 100% when tested with clinical samples using aptamer-Ab ELASA. This easy, particular, and sensitive method may be utilized as a point of care (POC) test near the patient, as well as a quick and early diagnostic tool. According to the findings of this investigation, the developed aptamer can be employed in a variety of aptamer-based fast detection techniques for the detection of CCHFV [135].

Fiber-optic biosensors (FOB) are a feasible substitute for traditional CCHFV detection approaches because of their small dimensions, high diagnostic precision, and relatively inexpensive. Other POC methods, including lateral flow, offer comparable benefits, but they do not provide measurable outcomes and have restricted multiplexing options. Because optical fiber biosensors are quantitative and easily multiplexed, they may be used to build a multiple-target POC test. Immobilization of captured affine biomolecules (for example, antigen, Ab, NA) at the end face and tip of an optical fiber is fundamental to creating optic-fiber biosensors. After that, the captured biomolecules bind to the target molecules, which can connect to supplementary tagged diagnosis biomolecules. When the indicator enzyme is exposed to a substrate, it oxidizes, producing a chemiluminescent glow that is accumulated through the optical fiber and relayed to the detectors [136].

The goal of Fairoz Algaar’s study is to develop a FOB for the exact diagnosis of Ab against CCHFV virus NP, as well as to modify fiber-optic immobilization techniques to increase the overall signal. They developed a FOB for detecting CCHFV IgG Ab. To enhance sensitivity, they tweaked both the bioreceptor immobilization technique and the chemiluminescence substrate composition. Finally, the FOB was tested throughout its tests with two CCHFV patient samples. Fairoz Algaar et al. showed that the FOB is tenfold more sensitive than colorimetric ELISA and can diagnose patients with maximum and minimum IgG Ab rates. They believe that the FOB is a better option than ELISA because it is much more sensitive and enables the diagnosis of a small number of Ab at an initial phase of infection. It may be used as a CCHFV POC detection method [137]. One strategy for obtaining rapid detection is to use molecular detection techniques on “lab-on-a-chip” strategies that handle and analyze therapeutically related biological or chemical samples in minute quantities. Optofluidic devices with the potential for biosensing, bioanalysis, and other uses are constructed by combining photonic principles into microfluidic chips. Many fascinating gadgets employ unique photonic concepts to develop practical POC mechanisms [138, 139].

Alexandra M. Stambaugh and colleagues explore an anti-resonant reflecting optical waveguide-based biophotonic analysis platform (ARROWs). ARROWs with orthogonally crossed solid-core ARROWs and analyte-carrying liquid-core ARROWs prepare for planar fluorescence stimulation and diagnosis of single biomolecule input. Furthermore, these silicon-based chips may be combined with improved sample formation stages on specialized microfluidic chips, enabling complete sample-to-answer molecular biomarker analysis in a chip-based system. They show how to use multimode interference waveguide technology to detect four different VHF entire RNA samples (Marburg marburgvirus, Ravn virus, and CCHFV) without amplification, as well as combinatorial fluorescence tagging of target NA. This notion is supported by the detection of single molecules in the total RNA sample after heat-triggered discharge from the transporter microbeads [139]. The researchers by utilizing an electrochemical reduction approach and the self-assembled monolayer (SAM) method, developed a 4-aminophenyl functionalized gold electrode (AuE) modified with silver nanoparticle-based graphene oxide (AuE-AP-GO-Ag). 4-nitrophenyl covalently to the surface of the AuE with its diazonium tetrafluoroborate salt by electrochemical diazonium reduction technique and 4-nitrophenyl modified glass carbon electrode was exposed to make 4-aminophenyl functionalized AuE (AP-AuE). The AP-AuE surface was treated with graphene oxide nanorobots connected to silver nanoparticles through the SAM process after a cathodic capability scan to decrease the nitro to an amine group. They used X-ray electron spectroscopy (XPS), infrared (IR) spectroscopy, cyclic voltammetry (CV), and electrochemical impedance spectroscopy to identify AuE-AP-GO-Ag (EIS). The nanomaterial was employed as a SERS form to detect ODN-based CCHFV using a DTNB Raman label linked to silver nanoparticles [140].

The ability of nucleic acid aptamers to bind viral envelope proteins has long been demonstrated, inhibiting the spread of deadly virus infections. John G Bruno et al. advanced and screened DNA aptamers versus recombinant envelope proteins or synthetic peptides, as well as entire deactivated viruses from a variety of virulent arboviruses, such as Chikungunya, CCHFV, dengue, tick-borne encephalitis, and West Nile viruses. To further establish their economic viability, the maximum desire and best-investigation CCHFV aptamers were employed in constructing a fluorescent aptamer–MB sandwich test. Other aptamer combinations were examined to identify the best one for the trial, and the Gn6-25R aptamer as a capture agent and the Gn6-17 F-TYE665 as a reporter gave better fluorescence detection. Aptamers scored well in the LF and fluorescent sandwich tests, indicating that they might be employed to detect arboviruses in these conditions. Some of the found aptamers can bind viral envelope proteins in vivo, making them antiviral in passive immunity or preventive applications. As a result, they’ve been made available to scientists for future research into diagnostic tests, biosensor applications, passive immunization, and prevention against dangerous infections [141]. Zgün Köse et al. present the creation of cryogel columns made by adenosine triphosphate (ATP) with ruthenium hapten cross-linker-based photosensitive technique “ANADOLUCA” for the biotinylation of CCHFV primers through the 5′ end. Without an ATP ligand, constant and quantum dots (QDs)-based avidity nanoparticles attached with streptavidin were employed to identify CCHFV. P (HEMA-co-DNA ligase) photosensitive copolymerized cryogel column with photosensitive ruthenium chelate-based hapten cross-linker was created and described as a biotinylated sustained method employing ATP ligand and photosensitive ruthenium chelate based hapten cross-linker. The resulting column’s biotinylated yield was then demonstrated by continuous biotin binding without requiring an ATP ligand to CCHFV. PCR was used to test the effectiveness of the biotinylated CCHFV primer. A sustained biotinylating column created from the identical ligand that they made utilizing DNA ligase while working with ATP in the DNA ligase biotinylation process can biotinize without ATP. Furthermore, when avidity interaction with the QD composition for viral detection was found following biotinylation of virus-identifying ssDNA, fluorescence was seen. The fluorescence intensity variations caused by the hybridization of 3′-ssDNA complements were then determined. The ANADOLUCA method for biotinylation and avidity-based identification of CCHFV primers [142] (Table 3).

**Table 3:** Biosensocrs for the detection of CCHFV

Method type	Detection target (segment)	LOD	Characteristics	REF
Aptamer-antibody ELASA test	Nucleoprotein (NP)	90 ng/ml	This easy, particular, and precise method can be used as a fast and initial diagnostic tool, as well as a POC test near the patient.	[135]
FOB	IgG Ab	Antibody dilution 10^− 6^ (200 µl diluted Ab)	The FOB is tenfold more sensitive than a colorimetric ELISA and can identify both high and low rates of IgG Ab in patients.	[137]
Anti-resonant reflecting Optical Waveguides (ARROWs)	RNA	240.3 ng/µL for MARV and 231.4 ng/µL for EBOV	The fluorescence signal amplitudes in the Ebola whole RNA sample were eight times higher than in three other whole RNA samples: Lake Victoria Marburg Virus, Ravn Marburg Virus, and CCHFV.	[138]
Surface-enhanced Raman spectroscopy (SERS)	RNA	-	The AuE-AP-GO-Ag NPs were employed as a SERS platform to detect ODN-based CCHFV using silver NPs connected with a DTNB Raman label. The calibration curve was obtained using the Raman shift of nitro label on a pM-sensitized nanoplatform.	[140]

### CRISPR

A Clustered Regularly Interspaced Short Palindromic Repeats (CRISPR) RNA (crRNA) with a “spacer” sequence that correctly matches the target NA sequence stimulus triggering of a CRISPR effector protein (Cas13a, Cas12a, or Cas12b), resulting in lateral cleavage of RNA or DNA reporters and considerable signal augmentation. This crRNA design technique may be utilized to identify CCHFV at the SHERLOCK technology’s Cas13a base (high-sensitivity enzyme reporter unlocking). In 30–40 min, it detects one copy/µl of vRNA from all clades of CCHFV strains, as well as newly reported strains with new alterations in the CRISPR target region. It also has no cross-reactivity with any of the CCHFV-related viruses. The CRISPR diagnosis based on degenerate sequences is a potential approach for successfully identifying extremely changeable viral infections. To understand more about the assay’s limit of detection (LoD), consider that RT-PCR has been the most broadly utilized molecular technique for identifying CCHFV genomic sequences thus far. The LoDs for these assays are generally in the 5–1000 cp/reaction range, with an average of around 10 cp/reaction. The LoD of the degenerate-sequence CRISPR test appeared comparable to that of the RT-PCR assay at roughly 25 cp/reaction [143].

### Potential of Novel Biomarker as Detection Agents

MicroRNA (miRNA) is a naturally occurring short non-coding RNA (ncRNAs) that controls the post-transcriptional expression of coding genes. All biological regulatory systems—cell differentiation, proliferation, immune responses, development, and death—are involved. Additionally, miRNAs may direct host reactions or encourage the spread of illness following infection [144]. Thus, miRNAs may represent promising new targets for developing diagnostic tools for a wide range of infectious disorders. Studies have shown that miRNAs may be employed as biomarkers and biosensors for diagnosing illness [145, 146]. ncRNAs larger than 200 nucleotides are long non-coding RNAs (lncRNAs), a diverse class of regulatory RNAs generated by specialized transcriptional units. Structured RNA molecules are frequently the product of LncRNA transcriptional units; these molecules interact with other biomolecules, including DNA, RNA, and proteins, and function as scaffolding for high-molecular-weight complexes with regulatory actions that impact genomic output at several levels. During the last decade, scientists have uncovered evidence that lncRNAs play essential roles in viral infections, either as drivers or mediators. LncRNA mediators have a major role in controlling the immune and inflammatory response to viral infections. These well-described cases define the regulatory role of certain lncRNAs during RNA virus infections [147, 148]. The signs and symptoms of VHFs also seem to be mostly or solely the result of the virus-infected monocytes’ and macrophages’ secretion of cytokines, chemokines, and other proinflammatory mediators. One of the initial research on cytokines in CCHF revealed that among 16 PCR-positive CCHF patients, the fatal case had the highest level of IL-10 and the highest amounts of TNF-α, IL-6, and other cytokines [149].

In this context, miRNAs constitute a potential marker. MiRNAs are short, endogenously generated RNA molecules (18–22 nucleotides) which bind to the 3′-UTR of target mRNAs to suppress gene expression. Numerous biological functions, including the control of the cell cycle, apoptosis, cell proliferation, and differentiation, depend on miRNAsIt has been discovered that miRNAs secreted from virus-target cells regulate viral infection and replication and that viral factors regulate intracellular miRNA expression. It is hypothesized that the virus causes inflammation and a breakdown of the vascular barrier by interfering with critical miRNA-regulated activities in endothelium cells, which are responsible for maintaining vascular health [32, 150–153]. According to research, miRNA expression is associated with meaningful communication in patients with CCHFV. As a result, diagnosing miRNAs in CCHFV patients will let the definition of therapeutic agents in infections. In this investigation, researchers evaluated miRNA expression in case-control, deadly-control groups, and fatal-nonfatal groups. Considerable miRNAs related to mortality were discovered in CCHFV. In addition, researchers utilized a microarray method forming a tremendous powerful, and affordable technique to screen B-cell peptide epitopes including the whole GPC of CCHFV. Significant miRNAs linked to mortality in CCHFV were identified in the research. In this investigation, they employed a microarray methodology to screen for B-cell peptide epitopes that cover the precursor of the entire CCHFV glycoprotein using an effective technique. This is the first research in South Africa and Turkey to employ microarray technology for screening clinical specimens in survivors of CCHFV infection [154]. miRNAs are having an increasing influence on the various viral infection pathways [155]. In the transition from the acute phase to recovery in CCHFV patients, the effects of miRNA through microarray have been investigated for the first time. Investigators shown that miR-15b-5p and miR-29a-3p were statistically substantially downregulated, but miR-4741, miR-937-5p, miR-6068, miR-7110-5p, miR-6126, and miR-7107-5p were upregulated in severe instances compared to convalescent patients. Whereas miR-6732-3p, miR-4436b-5p, miR-483-3p, and miR-6807-5p showed the most downregulation, miR-532-5p, miR-142-5p, miR-29c-3p, and let-7f-5p showed the greatest upregulation in acute patients compared to mild instances. As a result, CCHFV-induced miRNAs have been related to antiviral and proinflammatory pathways in both mild and severe patients. miRNAs may be a feasible target for diagnosis and therapy due to changes in their expression [156]. Ferraris et al. observed a different circulating miRNA (c-miRNA) profile in CCHFV patients’ IFN-receptor-deficient (IFNAR) KO mice compared to resistant wild-type (wt) animals. The plaque assay and qRT-PCR were used to quantify CCHFV. In this reaction, twenty c-miRNAs were found to be significantly changed, such as miR-122-5p, miR-216a-5p, miR-217-5p, miR-29a-3p, and miR-511-5p. Applying logistic regression examination, a mixture of eight miRNAs permitted a 100% differentiation of mice suffering an acute disease (IFNAR-KO) from unrecognizable clinical symptoms. It is uncertain whether a similar trait may be noticed in humans. However, further study is needed to assess if c-miRNAs might be unique, therapeutically significant prognostic instruments [157]. Compared to the control group, people with CCHF had less miRNA-126 and other miRNAs being expressed. The transcript of miRNA-126-3p, on the other hand, dropped by 45-fold. This is because it plays a part in making IFN-I and adjusting the mechanism of the immune response in patients [154, 158]. In the domain of miRNA detection, nanomaterials-based biosensing strategies offer several advantages over conventional diagnostic methods, including signal amplification, improved simplicity and sensitivity, and a flexible biosensing scheme that can be customized to target a specific interest. By integrating fluorescence nanotechnology techniques with QDs, scientists are capable of rapidly, efficiently, directly (cDNA is not required), inexpensively, sensibly, and precisely detecting miRNAs. Therefore, researchers examined the most recent developments in detecting miRNAs using fluorescent methods based on nanomaterials, including AuNPs, AgNCs, GO, MNPs, and SiNPs. It has been shown that the utilization of these nanomaterials can enhance the effectiveness, precision, and sensitivity of nanotechnology detection methods that are comparable to QDs. Although there are still limitations to their practical implementation as routine systems in clinical diagnostic and prognostic, advancements in nanobiotechnology and nano-bioscience offer a positive outlook for the utilization of nanomaterials-based fluorimetric approaches in the detection of miRNA [32, 159].

Long noncoding RNAs (lncRNAs) control gene expression in a variety of biological activities. For the first time in CCHFV, researchers analyzed the lncRNA gene expression patterns using a microarray. To validate the microarray results of several lncRNAs, a qPCR was used. Compared to controls, FER1L4, ECRP, and LOC100133669 are significant lncRNAs in both case and fatal case groups [160]. The CCHFV microarray comprises recombinant proteins NP and NPSH, antigenic regions of glycoprotein G1, and one protein L. The goal was to progress and assess a recombinant protein-based microarray for detecting IgM/IgG Ab against CCFV antigens. Microarray detects IgM/IgG distinctly but in one well because of the use of Cy3/Cy5-labeled particular Ab. The researchers demonstrated that the recently advanced microarray is a very accurate and specific diagnostic instrument [161].

The purpose of this research was to establish a correlation between early serum DcR3 levels and the clinical severity of CCHF in patients. A control group of forty healthy individuals and 88 patients hospitalized for CCHF between April and August 2022 were included in the study. Based on their clinical course, the patients were categorized as having mild/moderate (group 1, *n* = 55) or severe (group 2, *n* = 33) CCHF. The ELISA was utilized to quantify DcR3 levels in serum samples collected at diagnosis. Significantly higher incidences of hypoxia, fever, hemorrhage, nausea, headache, and diarrhea were observed in patients with severe CCHF compared to those with mild/moderate CCHF. Serum DcR3 concentrations were more significant in Group 2 than in Group 1 or the control group. Additionally, group 1 had substantially elevated serum DcR3 levels compared to the control group. Serum DcR3 had 99% sensitivity and 88% specificity in distinguishing patients with severe CCHF from those with mild/moderate CCHF when 98.4 ng/mL was used as the cutoff value. In contrast to other infectious diseases, CCHF can manifest a severe clinical course during the peak season in our endemic region, irrespective of age and comorbidities. Early detection of elevated DcR3 may permit the trial of additional immunomodulatory therapies in CCHF, a condition with limited treatment options beyond antiviral therapy [55].

### Limitations and Advantages of Novel Detection Method in CCHFV

Given the wide genetic diversity among CCHFV strains, molecular approaches that detect all of the virus’s currently known genetic lineages must be prioritized since the appearance of CCHFV strains from additional lineages in unexpected environments is relatively unusual. In acute conditions, Ab generation may be delayed or nonexistent. In CCHFV diagnosing laboratories, quality control tests are critical. There is a need for standardized assays and POC testing with excellent effectiveness. Early detection of CCHFV instances requires awareness, preparation, and monitoring [162]. Microarrays and other high-end molecular platforms take a long time, cost a lot of money, and generate much data. As a result, molecular-based techniques’ future potential should be focused on creating rapid, user-friendly, cost-effective, and portable technologies to prevent a sudden spike in prevalence worldwide, particularly in resource-constrained areas [163]. Furthermore, due to a lack of minimal laboratory equipment in many prevalent areas, the availability of viral culture as a detection instrument is usually unattainable [164]. Diagnostics currently use real-time RT-PCR and ELISA to identify Ab and antigens. These methods need specialized staff and costly tools, and they are not suitable for POC diagnostics. Furthermore, there is no point-of-care testing available for CCHFV. The first diagnosis uses PCR on blood, followed by a serological test [137].

Furthermore, no POC tests for CCHFV are available. Today’s diagnostic approaches in laboratories or diagnostic centers require expensive equipment or qualified personnel to operate; nevertheless, CCHFV-endemic regions are primarily rural, and diagnostic services in such places are limited. Because of CCHFV’s high pathogenicity and the high potential of CCHFV transmission from person to person, biosensors as rapid, reliable, safe, and high-sensitivity diagnostic tools might be extremely valuable in the diagnosis of CCHFV; however, further research is needed in this area [165, 166]. Biosensors, analytical instruments that use a bio-molecule as a recognition element, evolved in the previous decade for their speed and specificity, particularly intending to create a POC device. Biosensors are simple to use, do not require professional training, are portable, and offer data in a matter of minutes. Biomolecules such as Ab and NA have been extensively investigated and defined to ensure proper use in emerging technologies targeted at speeding up biological tests while preserving high specificity, such as biosensor applications [167].

## CCHF Infection Common Treatment Methods and Drugs

The existing therapeutic options for CCHF are limited. At present, the FDA has not approved any particular antiviral drug for the management of CCHF in people. Ribavirin, a potent antiviral medication, has shown exceptional potential over an extended duration. Furthermore, several articles have demonstrated the effectiveness of ribavirin in treating CCHFV infection in either oral or intravenous administration. Ribavirin, a nucleoside analog, has demonstrated antiviral efficacy against many HF viruses and other viral strains. Ribavirin, while lacking FDA approval for intravenous administration and being only licensed for oral use in the United States for treating hepatitis C, has been used for the management and prevention of CCHF, Lassa fever (LF), and hantaviruses. The primary basis for its utilization is derived from laboratory experiments assessing its efficacy and investigations done on animals. Nevertheless, the available information from human research is limited and sometimes relies on anecdotal accounts [168]. Also, D’Addiego et al. used next-generation sequencing to investigate the mutagenic effects of ribavirin on the genome of the CCHFV across the whole duration of the clinical disease. A study was performed on samples collected from CCHF patients who were solely receiving either ribavirin medication or supportive care at Sivas Cumhuriyet University Hospital in Turkey. The analysis of mutation rates across different groups provided little evidence for a mutagenic impact as a whole. This suggests that ribavirin, when given at the recommended dosage by the WHO during the first stages of CCHFV infection, does not have the potential to cause fatal mutagenesis. Fatal mutagenesis refers to the process of inducing mutations that would lead to the complete elimination of the CCHFV population and a decrease in the amount of virus in the bloodstream [169]. The efficacy of alternative therapies, such as Intravenous Immunoglobulin (IVIG), steroids, CCHF hyperimmune globulin, and CCHF mAbs, is still a topic of discussion [170].

Favipiravir, or T-705, is a nucleoside analog employed as a broad-spectrum antiviral agent that exhibits prospective activity against CCHFV. Favipiravir has been shown in vitro and in vivo to inhibit the synthesis of viral genomes by various RNA viruses, including SARS-CoV-2, Ebola virus (EBOV), Influenza virus, and Lassa virus. Two studies successfully established the beneficial impact of favipiravir in mice with advanced CCHF disease by employing mice as a model organism. The results of an in vitro investigation suggest that the concurrent administration of ribavirin and favipiravir may have a synergistic impact on CCHFV. This may indicate that the simultaneous administration of ribavirin and favipiravir at modest concentrations may reduce the likelihood of adverse effects while producing beneficial outcomes [150, 170, 171].

Ferraris et al. have assessed the potential antiviral efficacy of several compounds that target the entrance mechanism of CCHFV. Two compounds, namely chloroquine and chlorpromazine, were discovered by researchers. Neutralization and viral yield reduction tests were conducted on two distinct strains of CCHFV using Vero E6 and Huh7 cells. Multiple combinations, including ribavirin, were analyzed to evaluate a possible synergistic impact. Both chloroquine and chlorpromazine demonstrated inhibition of CCHFV. The specific values for the 50% inhibitory concentration (IC50) varied depending on the virus and cell lines used, with chloroquine ranging from 28 to 43 µM and chlorpromazine ranging from 10.8 to 15.7 µM. Time-of-addition tests revealed that these compounds had a direct impact on the infectivity and dissemination of CCHFV. The antiviral efficacy of the two compounds remained potent even when administered up to 6 h after infection and up to 24 h after that. The range of the selectivity index, which varies from 3 to 35, prompted us to assess combinations, including ribavirin. The combination of ribavirin and chloroquine or chlorpromazine had a synergistic effect against CCHFV. While the poor chlorpromazine selectivity index indicates the need for chemical enhancement, our current analysis emphasizes chloroquine as the primary medication with the potential for repurposing [38].

Severe CCHF, similar to other HFs, is characterized by an uncontrolled inflammatory response and excessive release of cytokines, resulting in significant immune system damage. Therefore, there have been little efforts to use anti-inflammatory medications in patients with CCHF to mitigate the excessive inflammatory reaction of the host. A comparative analysis of patients diagnosed with CCHF revealed that the administration of high-dose methylprednisolone in combination with ribavirin resulted in better results compared to the use of ribavirin alone. Evidence suggests that corticosteroids may provide advantages for people with severe illness. Nevertheless, the number of participants in these investigations is restricted. Recent research conducted on mice with blocked IFN-I and deadly infection showed that animals missing the TNF receptor or receiving therapy with an Ab to inhibit TNF signaling were able to avoid fatal illness. It may be necessary to assess the potential of this method to treat CCHF, considering the existence of clinically authorized TNF therapies and treatments targeting other host cytokines [2, 53, 61, 173].

## CCHF Infection Novel Treatment Methods

Currently, Gc is the only identified target of CCHFV-neutralizing Ab. According to reports, Gc remains monomeric during the prefusion stage and transitions to a trimeric state to facilitate membrane fusion in an acidic environment. CCHFV enters target cells by receptor-mediated endocytosis, and the fusion of the virus with endosomes occurs at multivesicular bodies. Nevertheless, the specific cellular receptor(s) responsible for CCHFV infection has not yet been identified, impeding our comprehension of the interaction between CCHFV and its host, as well as hindering the development of efficacious therapeutic approaches for CCHF. The present investigation revealed the LDLR as an essential entry receptor for the infection of CCHFV. Through the use of biochemical, cellular, and genetic methodologies, we provide evidence that CCHFV Gc has a direct interaction with LDLR. This interaction is capable of facilitating the entrance of CCHFV into cells of both mouse and human origin, as well as enabling the establishment of infection and the development of disease in mice [42].

An investigation included creating a minigenome system for orthonairoviruses, namely CCHFV and Hazara virus, to examine viral replication. Additionally, a library of FDA-approved compounds was examined. Introducing the minigenome components by transfection resulted in a significant augmentation in luciferase expression, suggesting that the reporter RNA underwent adequate replication and translation. Through the process of compound library screening, a total of 14 candidate compounds were discovered. These compounds were shown to have a substantial impact on reducing luciferase activity. Certain chemicals also hindered the proliferation of the pathogenic Hazara virus. An investigation was conducted to evaluate the inhibitory mechanism of tigecycline. It was discovered that tigecycline caused a reduction in the interaction between the viral N protein and RNA. This study establishes a foundation for validation via animal models and the creation of chemical compounds that exhibit enhanced activity. These findings will be valuable for future research on developing an antiviral treatment for CCHFV [174]. Both the virus and the host cell might have different viewpoints on the role of cholesterol in viral infection. The only thing that matters for a virus particle is its ability to get enough sterol from the infected cell to maintain the structure of its envelope membrane bilayer since it does not have any internal membrane structures. Therefore, broad-spectrum antivirals might potentially improve the health outcomes of patients and expedite the therapy of viral infections. miRNAs, which are small RNA molecules that regulate gene expression after transcription, have been recognized as significant regulators of lipid homeostasis. These discoveries reveal the intricate complexity of lipid balance in the body and the vital role that miRNAs play in controlling this process. This suggests new strategies for treating viral infections. Further investigation is required to understand the role of miRNAs in regulating cholesterol balance, particularly in the context of cirrhosis and the chronic inflammation associated with viral hepatitis [175, 176].

U18666A, a cationic amphiphilic molecule, has been shown to block Niemann-pick C1 (NPC1), a cholesterol transporter, therefore effectively impeding the egress of cholesterol from lysosomes. U18666A is a pharmaceutical agent that inhibits viral infection and impacts both the fusion and replication stages of the life cycles of different viruses. Collectively, the results suggest that U18666A has potential as a therapeutic agent for combating viruses by impeding their transition from early and late endosomes into lysosomes after endocytosis, hence hindering their ability to replicate. While the in vitro findings discussed above provide evidence for the important role of lipid rafts and cholesterol in viral entry, conclusive verification in vivo is necessary. The potential significance of lipid rafts in aiding the cellular entry of coronaviruses suggests that new therapeutics for SARS-CoV-2 might be developed. Further investigation is required to determine the viral and cellular factors that contribute to the reduction of viral infection by U18666A treatment. To enhance researchers’ comprehension of the interaction between viruses and host cells, as well as to facilitate the development of novel therapeutic approaches, it is essential to get a more comprehensive understanding of the impact of U18666A-induced changes in intracellular cholesterol balance on viral infections. This medication’s antiviral capabilities are so promising that it justifies thorough clinical testing. Hence, investigators advocate for using nanoparticles for precise therapeutic interventions, alongside more investigations to ascertain the adverse effects of this medication and the optimal dosage for various viral illnesses [177–179]. Investigating the impact of lipids and cholesterol on CCHFV may contribute to the advancement of therapeutic approaches.

Utilizing metabolomics to investigate alterations in the metabolism of hosts caused by viral infections has demonstrated to be a revolutionary methodology. Before Gocenler et al. research, there was a solitary ‘omics’ study that examined the interaction between the host and CCHFV, as well as the consequent pathogenesis of the virus. They utilized NMR spectroscopy due to its prowess in analyzing complex biofluids, minimal sample preparation requirements, and reproducibility. The approach researchers employ in this study is founded on the established achievements of metabolomics in investigating VHFs, including Ebola, Marburg, and Dengue. Researchers’ research emphasizes the pivotal significance of SAH, a metabolite that participates in many biochemical reactions. Also, investigators offer novel perspectives on the metabolic changes in patients with CCHF. These modifications provide insight into the pathogenesis of the disease but also establish a foundation for potential biomarkers and therapeutic targets. S-Adenosyl-L-homocysteine and Carnosine emerged as the most abundant metabolites among those identified, thus justifying additional investigation into their involvement in the pathogenesis of CCHFV and their potential as targets for therapeutic interventions [180]. An image-based phenotypic high-throughput screening test, including automated image processing, was created by Tampere et al. to assess a set of tiny pharmacological inhibitors that were internally generated and focused on nucleotide metabolic pathways and oxidative stress. The antiviral compounds TH3289 and TH6744, which were recently found, have a broad spectrum of antiviral actions against many RNA viruses, including CCHFV, EBOV, Hazara virus, and SARS-CoV-2. HSP70-related cellular pathways are probably affected by these impacts [181].

### Immunotherapy in CCHFV

Based on research findings, upon viral entry into the body, immune cells, including dendritic cells and macrophages, are compromised, and inflammatory cytokines (e.g., TNF-alpha, IL1, and IL6) are secreted. These cytokines generate cytokine storms, which induce shock through endothelial activation and vascular leakage. Conversely, the formation of clots and DIC leads to extensive impairments in multiple organs, including the liver and kidneys. The importance of disease prevention and treatment cannot be overstated, given the absence of an alternative efficacious vaccination. As a result, immunotherapy is employed. When integrated with compensatory therapies, including but not limited to Fresh Frozen Plasma (FFP) replacement, hydration, electrolytes, and blood and platelet replacement, one of the most efficacious regimens is maintained. Research has demonstrated that certain anti-inflammatory treatments, such as corticosteroid-based immunotherapy, neutralizing and non-neutralizing mAbs, cytokine therapy, and IVIG immunotherapy, are efficacious in managing the disease [182]. Ab that neutralize viruses represent a potentially effective treatment for infectious diseases. In this investigation, 37 specific mAbs against the CCHFV Gc subunit were produced from mice. Ab that neutralized pseudotyped viruses and authentic CCHFV were identified as Gc8, Gc13, and Gc35 in neutralization assays. Gc13 exhibited the most excellent affinity for and neutralizing activity against CCHFV Gc. In a lethal mouse infection model, Gc13 consistently demonstrated in vivo protective efficacy (62.5% survival rate) against CCHFV infection, while neither Gc8 nor Gc35 did so. Gc8 and Gc13 may recognize a similar, linear epitope in domain II of CCHFV Gc, whereas Gc35 may identify a distinct epitope in Gc, according to additional characterization studies. The analysis of Gc-Fab complexes via cryo-electron microscopy revealed that Gc8 and Gc13 bond to the conserved fusion loop region, with Gc13 exhibiting more robust interactions with sGc-trimers. This was substantiated by the fact that Gc13 inhibited the GP-mediated membrane fusion of CCHFV. In general, this research offers novel therapeutic approaches for the management of CCHF and provides fresh perspectives on the way Ab interacts with CCHFV Gc proteins [183]. An increasing body of scientific literature underscores the importance of the IL-36 family as a component of the proinflammatory signaling pathway. To date, however, the potential of IL-36 family members as biomarkers in CCHF has not been investigated. By comparing the levels of IL-36α, IL-36β, and IL-36γ in healthy controls and patients with CCHF, this study attempts to close this knowledge gap by examining the correlation between these biomarkers and disease severity and prognosis. In this case-control investigation, 60 confirmed CCHF patients and 29 healthy controls were enrolled. IL-36γ, IL-36β, and IL-36γ concentrations in the serum were determined using ELISAs. CCHF patients exhibited notably elevated concentrations of IL-36α and IL-36β compared to the control group of healthy individuals. Nevertheless, there were no IL-36γ levels that differed significantly from one another between the two groups. A significant increase in IL-36α and IL-36γ levels was observed in non-survivors of CCHF compared to survivors. IL-36α and IL-36γ levels positively correlated with activated partial thromboplastin time and D-dimer. On the contrary, platelet counts exhibited an inverse relationship with both IL-36α and IL-36γ concentrations. Patients with CCHF have elevated levels of IL-36α, IL-36β, and IL-36γ, which suggests that these molecules are involved in proinflammatory responses. Understanding the involvement of IL-36 family members in the pathogenesis of CCHF may provide significant knowledge regarding the advancement of the disease and aid in the formulation of precise therapeutic approaches [184].

Golden et al. conducted a study utilizing adult mice to examine the efficacy of glycoprotein-targeting neutralizing and non-neutralizing mAbs in protecting against CCHFV infection. Adult IFN-I-deficient mice were protected by a solitary non-neutralizing Ab (mAb-13G8) to greater than 90% when treatment commenced before virus exposure and greater than 60% when administered after viral exposure. Immunoglobulin G subclass notwithstanding, neutralizing Ab that are known to protect neonatal mice from fatal CCHFV infection were ineffective in achieving this. mAb-13G8 was found to target GP38, which is among several GP derived from the CCHFV GPC polyprotein that is cleaved proteolytically. This research establishes GP38 as a critical Ab target in impeding the progression of CCHFV and provides the groundwork for developing human immunotherapeutics against CCHFV [185]. It has been reported that mAbs that neutralize the glycoprotein Gc of CCHFV can protect mice from challenges with the prototype CCHFV strain, IbAr10200. However, the neutralization of other CCHFV strains by these mAbs remains unknown due to the considerable sequence diversity of CCHFV GP. Initially, a CCHF VLP system was used to create eleven VLP moieties, each containing a glycoprotein from a genetically distinct CCHFV strain obtained in Africa, Asia, the Middle East, or southeastern Europe. These VLPs were used by researchers to effectively test the cross-neutralization efficacy of mAbs under biosafety level 2 settings. Most CCHF VLPs showed cross-neutralization activity with three mAbs (8A1, 11E7, and 30F7); 8A1 neutralized every VLP tested. Although binding tests show that none of the mAbs compete with one another for the same epitope, in this specific system, adding 11E7, 30F7, or both to 8A1 did not increase neutralization. Researchers validated their findings from the VLP system by confirming that five different CCHFV strains were neutralized in vitro by the three mAbs that were able to perform strain cross-neutralization. Escape mutants resistant to further neutralization were not produced when CCHFV strains were passaging in the presence of mAb concentrations below neutralization limits. The previous research demonstrates how well the VLP technique works to screen for and neutralize mAbs against various CCHFV strains. Additionally, it offers the first proof that a single mAb can neutralize a range of CCHFV strains in vitro. This discovery has the potential for the future development of CCHF treatments [186].

### Limitations and Advantages of Novel Treatment Method in CCHFV

The past publications mainly focused on various choices for supportive therapy, such as steroids and immunoglobulin, as well as the effectiveness of antiviral medications, particularly ribavirin and favipiravir. While several research has validated the clinical efficacy of ribavirin in treating CCHF, other investigations have failed to establish its usefulness. Multiple studies have consistently shown that supportive treatments constitute the fundamental cornerstone of therapy. Supportive treatment is the essential approach to managing CCHF. The effectiveness of ribavirin for treating CCHF is uncertain, and more randomized case-control clinical studies are necessary to validate its use and provide recommendations for CCHF therapy. Additionally, the efficacy of other therapeutic approaches such as steroid injection, immunoglobulin therapy, and mAbs need more definitive evidence. Favipiravir is a very promising antiviral medication for the treatment of CCHF [187].

Considering the protracted process of antiviral drug development and approval, one potential approach could be to repurpose established medications for use in different medical conditions. Extensive human dosing experience is available for the majority of these drugs. Furthermore, the profiles of their excretion, metabolism, safety, absorption, and distribution are well understood. It has been demonstrated that the clinical drugs chloroquine and chlorpromazine, which are used to treat non-viral diseases, inhibit CCHFV in vitro. Although viruses can vary considerably, their life cycles share several stages in common. These stages consist of entry, biosynthesis, assembly, and release. Viruses typically target host processes and proteins similar to their own to develop host-directed, broad-spectrum antiviral drugs. Virus mutation is a significant obstacle in developing antiviral drugs, particularly for the RNA virus CCHFV, which has a high mutation rate. Considering several impediments to advancement, it seems certain that CCHF will persist as a substantial menace to public health in the foreseeable future. Progress in fields such as the research of viral protein structures, the creation of animal models with a strong immune system, and the investigation of the interaction between viruses and hosts will facilitate the development of efficient treatment interventions against CCHF. Furthermore, achieving effective CCHF therapies necessitates cooperation across nations where the disease is prevalent. Additionally, a model using cynomolgus macaques with a fully functioning immune system has been created and used to assess the effectiveness of favipiravir therapy against CCHFV infection. Further experimentation and refinement are necessary to evaluate the effectiveness of countermeasures against CCHFV using this non-human primate model. This includes doing experiments with more suitable delivery methods and virus doses. Enhanced comprehension of virology and the interactions between viruses and hosts in the future could yield valuable insights for the creation of engineered animal models. These models would possess specific virus-infection-associated host factors that are humanized, offering substantial benefits over models where common immune signaling proteins are merely removed. To further the study of medicines for CCHFV, it is necessary to establish more suitable animal models for pre-clinical research since the existing assessment of anti-CCHFV medications has been confined to animal models and clinical trials [37, 188, 189]. It is indisputable that CCHF will continue to pose a substantial public health risk for the foreseeable future, given the numerous impediments to progress. Additional progress in domains including the structural analysis of viral proteins, the development of immunocompetent animal models, and the investigation of virus-host interaction would facilitate the formulation of efficacious medical countermeasures against CCHF. Further, collaboration among endemic nations should be an integral component of successful CCHF therapeutic research. Furthermore, an immunocompetent cynomolgus macaque model was established and employed to assess the effectiveness of favipiravir treatment in combating CCHFV infection. Further evaluation and optimization of countermeasures against CCHFV should be conducted on this non-human primate model. This can be achieved through experimental settings that utilize more suitable administration approaches and viral dosages. In the future, a more comprehensive comprehension of virology and the interactions between viruses and hosts could yield fresh insights that could facilitate the creation of engineered animal models featuring humanized virus-infection-associated host factors. These models are anticipated to offer substantial benefits over those lacking common immune signaling proteins. Existing clinical trials and animal models for evaluating anti-CCHFV therapies are limited in scope; therefore, future research should concentrate on the development of more suitable animal models of CCHFV to facilitate preclinical investigations of therapeutics [37, 190, 191].

## Vaccination Method Against CCHFV

At this time, no licensed vaccines are accessible for CCHFV. The lack of an animal model susceptible to CCHFV infection significantly impeded vaccine development efforts. Despite succumbing to disease, newborn rodents are unsuitable for evaluating the efficacy of vaccines due to their lack of similarity to human CCHF and immature immune systems. To assess the effectiveness of vaccines, numerous animal models of CCHFV infection have been developed in recent years. Diverse strategies have been implemented to create a CCHFV vaccine. The vaccines encompassed in this category are those based on recombinant subunits, human adenovirus 5-vectored vaccine, virus replicon particle (VRP) vaccine, gene-based vaccine platforms (DNA or mRNA), vesicular stomatitis virus-based vaccine, and modified vaccinia Ankara–based vaccine [191–193].

### Nucleic Acid Vaccines

There aren’t many easily available vaccinations available right now, and even while the WHO recommends ribavirin as a therapy, its efficacy is yet unknown. This research evaluates a potential replicating RNA vaccine in a rhesus macaque (*Macaca mulatta*) model of CCHF. In place of the well-known cynomolgus macaque model, this alternative model replicates a range of mild to moderate human disorders. Persistent viremia, vRNA detection in several organs, and significant pathology in the liver and spleen of Rhesus macaques are the hallmarks of CCHFV infection. The model was used to evaluate the immunogenicity and protective effectiveness of a replicating RNA vaccine. After receiving an immunization with RNAs encoding the CCHFV NP and GPC, rhesus macaques showed a strong defense against the CCHFV challenge as well as a strong development of non-neutralizing humoral immunity against the NP. Researchers’ combined results demonstrate the immunogenicity and protective benefits of investigators’ replicating RNA vaccine in non-human primates after a prime-boost vaccination, and they also offer a model of CCHF using rhesus macaques [194].

Suschak et al. previously evaluated a DNA vaccine carrying the laboratory CCHFV strain IbAr 10,200’s M-segment GPC gene (CCHFV-_M10200_). Mice were protected by CCHFV-_M10200_ against a challenge with homologous CCHFV-IbAr 10,200 by more than 60%. In this study, it is documented that augmenting the dosage of CCHFV-_M10200_ in mice results in comprehensive immunity against challenges from homologous CCHFV and substantial (80%) protection against challenges from the clinically significant heterologous strain CCHFV-Afg09-2990. In addition, researchers document complete immunity against the CCHFV-_Afg09−2990_ challenge after administering a CCHFV-_Afg09−2990_ M-segment DNA vaccine. In conclusion, Suschak et al. demonstrate that GP38, a non-structural M-segment protein, exerts an impact on the immunogenicity of the CCHF vaccine and confers substantial protection against homologous CCHFV challenge. The findings of this research exemplify the protective immunity of CCHF that M-segment DNA vaccines induce, in addition to highlighting the immune relevance of GP38 [10].

Hawman et al. described how they successfully protected a non-human primate disease model from CCHFV-mediated infection. A DNA-based vaccine was administered to *Cynomolgus macaques* via in vivo electroporation-assisted delivery. The vaccine was composed of two plasmids, each encoding the NP and GPC of CCHFV. They observed robust T-cell and Ab responses in animals that had received three vaccinations. The NP + GPC vaccinated animals were considerably more resistant to CCHF-induced disease, viremia, and high tissue viral burdens than the sham-vaccinated animals. In summary, this supports the notion that a vaccine may offer protection against disease induced by CCHFV in a model of non-human primates. This facilitates vaccine clinical development for populations at risk of contracting the disease [195].

**Fig. 5:**
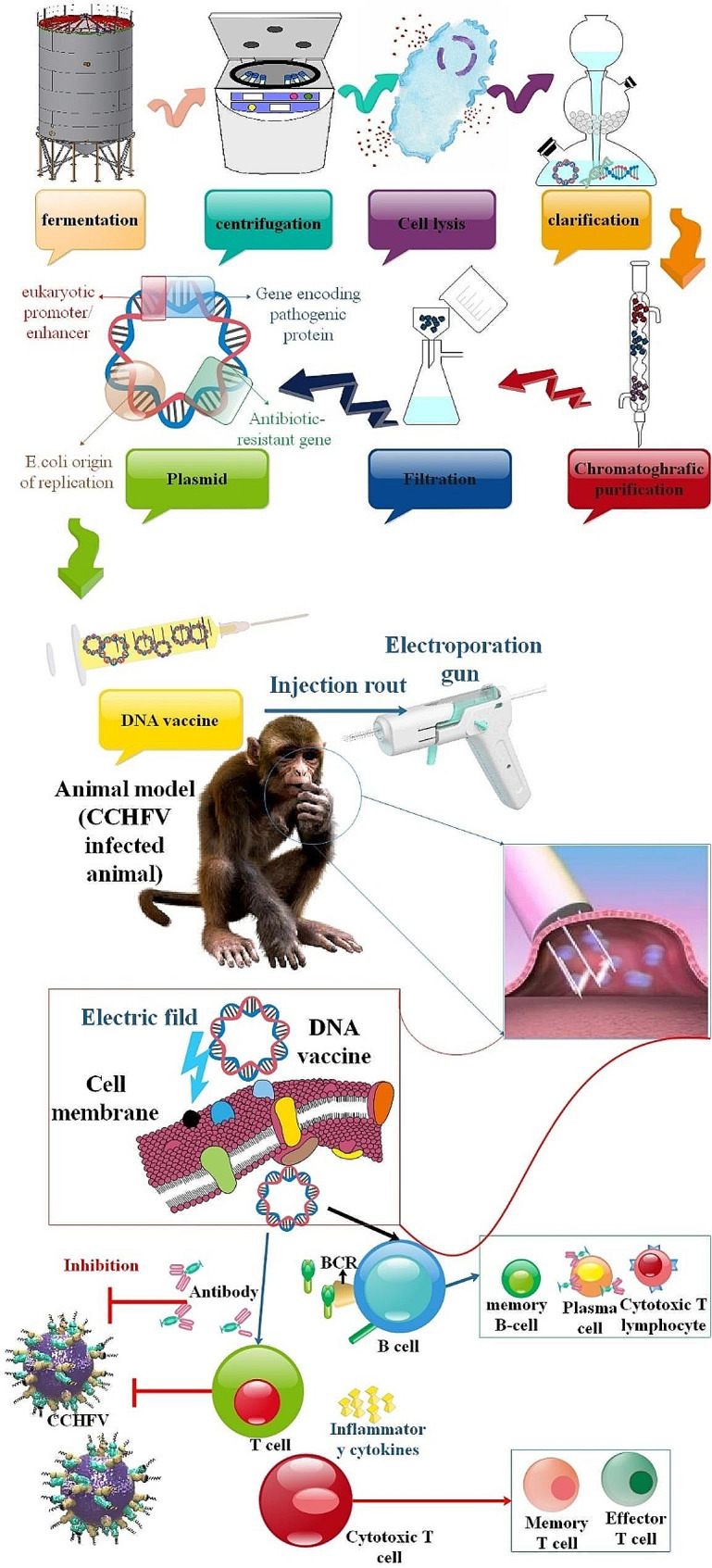
In a recent publication, scientists described the initial vaccine that exhibited effectiveness against CCHFV using a cynomolgus macaque model. A prime-boost-boost regimen involving in vivo electroporation of DNA plasmids encoding the NP (pNP) and GPC (pGPC) protected macaques infected with CCHFV significantly against viral replication and disease. Scholars present an advanced comprehension of the protective mechanism of this vaccine, as well as a significant refinement for the benefit of public health, in this article. Two immunizations of infected macaques with this vaccine platform containing both the NP and GPC antigens provide substantial protection against CCHFV, as demonstrated. It is noteworthy that they discovered that the combined administration of pNP and pGPC exhibited a higher level of protection against CCHFV infection compared to the use of three immunizations consisting solely of pNP or pGPC. Ab to antigens, humoral immunity, and cellular immunity may all contribute to vaccine-mediated protection against CCHFV, according to investigators’ findings [197]

In a previous study, Hawman et al. documented the noteworthy effectiveness of a three-dose DNA-based vaccination protocol against CCHFV in cynomolgus macaques (*Macaca fasicularis*). Here, researchers demonstrate that plasmid-expressed CCHFV NP and GPC antigens primarily induce humoral and cellular immunity, respectively, in cynomolgus macaques. A two-dose vaccination regimen containing plasmids expressing the NP and GPC provides substantial protection against CCHFV infection, according to their findings. Research examining vaccinations using plasmid-expressed NPs and antigen-only vaccines revealed the potential for protection. Researchers findings indicate that this vaccine provides comprehensive protection against CCHFV and that optimal vaccine-mediated protection is mediated by both humoral and cellular immunity [196] (Fig. 5).

mAbs that targeted the glycoprotein GP38, which is particular to that study, provided protection. Neutralizing Ab directed towards GC exhibits more significant variability in terms of protection. Thus far, only one engineered bicistronic Ab has demonstrated therapeutic efficacy. This Ab was created by combining two neutralizing Ab that target different sites on one of the six identified antigenic sites in GC. These results demonstrate the potential protective effects of extrinsically delivered Ab. The mean time-to-death of the CD8^−/−^ mice vaccinated with CCHFV-MAfg09 was significantly longer than that of the group vaccinated with an empty vector. This suggests that the Ab response may have offered some protection. This may indicate that vaccination-induced humoral responses against the GP fail to generate adequate levels of Ab against critical protective epitopes, thereby impeding the development of robust protection. In summary, this study demonstrates that the protective effectiveness of the M-segment-based DNA vaccine against CCHFV depends on the CD8 + T-cell response. Further research examining the immune response to each component across multiple vaccine platforms is required to ascertain whether our conclusions regarding GPC remain valid outside of DNA vaccination. Since antigenic strategies to target CD8 + T cells differ from a global immune system strategy, the insights garnered from these studies could be utilized to enhance the design of the GPC component of CCHFV vaccines. By using the IFN-I blockade paradigm in rodents devoid of particular adaptive immune compartments, our research establishes a framework for investigating such inquiries [197].

Studies have shown that the prototype of mRNA treatment, known as direct mRNA injection, induces the production of particular proteins that trigger an immunological response. RNA-based vaccines provide notable advantages compared to traditional immunization methods due to their high potency, potential for secure administration, cost-effectiveness in manufacturing, and ability to be rapidly developed. Reports indicate that mRNA-based vaccination elicits a more pronounced CD4 + or CD8 + T cell response than protein immunization [198, 199]. The efficacy and safety of the two most widely used mRNA vaccines against SARS-CoV-2 and its many variants have been shown in clinical studies [200]. Scientists provide evidence that immunization with nucleoside-modified mRNA-lipid nanoparticles (mRNA-LNP) containing the genetic information for the CCHFV NP or GP (GcGn) effectively shields IFNAR^−/−^ mice against fatal CCHFV infection. Furthermore, research shows that both mRNA-LNP formulations elicited robust humoral and cellular immunological responses in both IFNAR^−/−^ and immunocompetent mice. It was also shown that neutralizing Ab is not essential for providing protection. Upon assessing the immunological reactions triggered by vaccination with CCHFV Gc and Gn antigens, it was observed that the Gc protein exhibited higher immunogenicity in comparison to the Gn protein. Hepatic damage is common in CCHF and has a significant role in the severity and death rates of the illness in people. Therefore, to comprehend the immune response in the liver after infection and the possible impact of the vaccination, researchers conducted a proteome study on liver samples obtained from vaccinated mice and mice that served as controls after being infected with CCHFV. Consistent with findings in people, immunization had an impact on metabolic pathways. Overall, investigations demonstrate that a CCHFV mRNA-LNP vaccine, using viral nucleo- or GP, confers protection against CCHFV-induced illness. Hence, genetic immunization presents a compelling strategy for averting diseases caused by CCHFV. Investigators are confident that they possess the requisite data to advance this vaccine platform to the subsequent stage in creating a vaccine against CCHFV infection [201].

The current research included the creation of a novel mRNA vaccine without any protective covering, which expresses the non-optimized small (S) segment of the Ank-2 strain of the CCHFV. Researchers then examined the immunogenicity and protective capabilities of both the single and booster doses of the vaccine in two different mouse models, namely IFNα/β/γR^−/−^ and C57BL/6, using a challenge test. The outcomes derived from the immunological tests, including IL-4 and IFN-gamma ELISPOT, intracellular IFN-gamma staining, in-house sELISA, and survival data, clearly showed that their construct stimulated the generation of immune responses targeted against the nucleocapsid (N) in both mouse models. The booster dosage group of IFNα/β/γR^−/−^ mice achieved a protection rate of 100%, suggesting that more tuning is required for this platform in future investigations and ultimately, researchers evaluated a new method for vaccinating against CCHFV by using a traditional mRNA platform. This strategy shows promise for future research as an effective and secure means of combating this illness [202]. Researchers conducted a study using an immunoinformatics technique to create a universal mRNA-based multi-epitope vaccination called CCHF_GN728 for the CCHFV. The GPC and NP proteins from the virus were chosen and examined for possible T-cell and B-cell epitopes that may trigger an immune response. The antigen they created can provide global coverage to 99.95% of the population. The stability of the epitope-allele association was verified by the use of molecular docking and dynamics modeling techniques. The in silico immunological simulation confirmed that the immune cells responded to the pace at which antigens were cleared. Optimized codons guaranteed the mRNA was expressed efficiently in the host cell. The vaccination demonstrated consistent and robust interactions with the Toll-like receptors. The researchers’ results indicate that the CCHF_GN728 vaccine will elicit targeted immune responses against the CCHFV virus. Their model is prepared for wet lab experiments to evaluate the effectiveness of this potential vaccination candidate [203].

### Viral Vectored Vaccines

Modified Vaccinia virus Ankara (MVA)-based Poxviral vectored vaccines, for instance, can carry sizable gene inserts, including the CCHFV M segment. MVA can stimulate high-level gene expression of recombinant genes in vivo and in vitro despite its lack of proliferation in most mammalian cells. This is achieved by incorporating genuine post-translational modifications in the recipient cell. MVA is capable of stimulating both humoral and cellular immunity without the need for an adjuvant [193]. Of instances that are documented, 15–70% are deadly, and there is currently no licensed vaccination. A recombinant candidate vaccine expressing the CCHF virus NP was created in the past using the attenuated poxvirus vector, MVA. Two mouse strains, including IFN-I receptor deletion mice prone to CCHF illness, were tested for cellular and humoral immunogenicity. In a difficult scenario, the vaccine did not shield animals from deadly illness despite the immunological responses produced after vaccination. When challenged with the CCHF virus, the MVA-NP vaccine candidate showed antigen-specific immunogenicity in mice but no protective benefits. This indicates that vaccinations against the CCHF virus must show protection in a fatal dosage scenario before protective effectiveness can be demonstrated since there are no immunological correlates of protection [204].

In another study, an experimental CCHF vaccine vector was constructed and characterized by researchers. The vector was based on human adenovirus type 5 (Ad) and expressed the CCHFV N (Ad-N). Cell lysates contained detectable amounts of the CCHFV N protein after infection with recombinant Ad-N. Ad-N-immune mice developed a humoral immune response against N. When administered as a single dose of Ad-N, IFNAR-/- mice were protected against lethal CCHFV challenge by 30%. This could be increased to 78% protection through the use of a prime-boost regimen. N functions significantly as a protective component of a CCHFV vaccine, according to these findings. In summary, this study presents findings on the limited protective effectiveness of an Ad-based vaccine vector carrying the CCHFV N (Ad-N) against a lethal challenge of CCHFV in a mouse model that is immunocompromised and highly susceptible. This more conserved CCHFV antigen mediates a partial efficacy of up to 78% after a prime-boost vaccination strategy; this finding underscores the antigen’s pivotal function in safeguarding against CCHFV and implies its potential inclusion in future CCHFV vaccine strategies [192].

The preclinical evaluation of a chimpanzee adenoviral vectored vaccine (ChAdOx2 CCHF) that encodes the GPC from CCHFV is presented by Saunders et al. Here, they showed that immunization with ChAdOx2 CCHF results in 100% protection in a fatal CCHF challenge scenario and a humoral and cellular immune response in mice. The significant levels of CCHFV-specific cell-mediated, and Ab responses in mice are induced by administering the adenoviral vaccine in a heterologous vaccination regimen paired with the MVA CCHF. Further evidence of protection against illness is provided by histopathological inspection and viral load analysis of the tissues of ChAdOx2 CCHF immunized mice, which show no signs of microscopic alterations or viral antigens linked to CCHF infection. An efficient CCHFV vaccine is still required to shield people against the deadly hemorrhagic illness. Their results encourage the ChAd platform’s further development to express the CCHFV GPC and create a CCHFV vaccine [205].

Researchers detail the formulation and evaluation of a candidate vaccine comprised of CCHF VRP. It was postulated that the administration of VRPs, which resemble the composition and structure of genuine CCHFV, could potentially elicit a more pronounced establishment of CCHFV immunity compared to existing experimental vaccine alternatives. The procedure for generating VRPs is founded upon the reverse genetics system earlier elucidated for CCHFV strain IbAr10200. On the contrary, all surviving vaccinated animals (17/19) developed Ab against NP, while Ab against Gc was present in all but one animal (18/19). Mice vaccinated with the low dosage had significantly higher anti-NP and Gc Ab titers after CCHFV exposure. However, in mice that got the high VRP dosage, the titers of both NP and Gc Ab only slightly rose, which may indicate that CCHFV replication was absent or was at a lower level than in the animals who received the low dose. To determine their significance in protection, further research on T-cell responses and Ab activities will be required. Collectively, investigators’ findings validate the security and effectiveness of a solitary administration of this innovative vaccination in immunocompromised IFNAR^−/−^ mice, a highly susceptible CCHFV model. Reverse genetics systems are readily modifiable, and they might be used as a platform for developing novel CCHFV vaccines or to boost their effectiveness at a lower dosage possibly. It is uncertain what immune response to CCHF survival or experimental vaccinations are linked to. Without producing illness, VRPs mimic most of the processes involved in CCHFV replication. As such, they may serve as a helpful model for defining protective immune responses, all without raising the biosafety issues related to utilizing live CCHFV in experiments. In summary, researchers’ findings validate CCHF VRPs as a potentially effective vaccination platform that may lessen the risk of CCHFV to the public’s health. Future research should concentrate on better characterizing the immune response to immunization, determining the timing of vaccination (i.e., before and after exposure), and clarifying the protective mechanisms [206].

As experimental vaccines, recombinant vesicular stomatitis viruses (rVSV) that express foreign GP have demonstrated promise against several VHFs. In a study, a replication-competent rVSV vector encoding the CCHFV structural GP, CCHFV GPC, was constructed and evaluated. This construct induces robust in vitro expression of CCHFV-GP. Researchers vaccinated STAT-1 knock-out mice, which serve as an animal model for CCHFV, with these vectors. The vector was well tolerated and exhibited complete efficacy when tested against a clinical strain of CCHFV. Titers of neutralizing Ab and anti-CCHFV-GP IgG were detected in surviving animals. A rVSV expressing exclusively the CCHFV-GP has the potential to function as a replication-competent vaccine platform against CCHF infections, according to the findings of this study [207].

### Inactivated Vaccines

For more than a century, inactivated vaccines have been used to produce immunity against viral infections. Historically, vaccinations have been developed using purified inactivated viruses, and research has shown that these vaccines are both safe and efficient in preventing illnesses like the poliovirus and influenza virus [208, 209]. The conventional method of vaccine manufacture is reasonably easy to accomplish and offers an enhanced safety profile compared to live vaccines. Typically, all inactivated viral vaccines have a similar manufacturing process, whereby the pathogen is first grown on a substrate to generate substantial amounts of antigen. These terrible occurrences operate as a cautionary tale for all vaccine researchers. Simply deactivating a pathogen does not automatically result in a vaccine that can generate protective immunity. It is crucial to maintain the viral epitopes required to induce protective immunity following deactivation [210]. Vaccines against inactivated viruses are made from intact viruses that have been heated or chemically processed. Heat and chemicals like formaldehyde and phenol denaturate the surface proteins, rendering them inactive and non-infectious. Specific epitopes that remain unaltered after the whole virus is treated retain part of its integrity and trigger the production of Ab, triggering an adaptive immune response. The virus is divided into smaller antigenic pieces by phagocytic immature dendritic cells upon injection. These antigenic fragments are then exposed on the surface of MHC cells, activating B cells and T helper cells. There are no adverse side effects from inactivated vaccinations. Because they are incapable of replicating, they are heat-stable, noninfectious, and nontransmissible to the illness. Booster doses may sometimes be required to generate a significant immune response, however, since they are typically not as effective as live vaccinations and are insufficient as a single dose [211, 212].

In a study, the durations of the immune response, immunogenicity, and development of inactivated vaccine (CCVax) formulations derived from cell culture were compared to those of vaccine (MBVax) formulations derived from mouse brains. The Kelkit06 CCHF virus strain was cultivated in neonatal mice and Vero E6 cells for this investigation and subsequently isolated using a sucrose gradient. Various concentrations of formalin-inactivated vaccine candidates were formulated and combined with an alum adjuvant: low dose (LD), 5 µg; medium dose (MD), 10 µg; and high dose (HD), 20 µg. Three times every three weeks, identical doses of the vaccine formulations were administered to BALB/c rodents. The humoral endpoint IgG responses to the CCVax and MBVax treatments were compared and evaluated. Up to a year after vaccination, the duration of IgG and neutralizing Ab titers was assessed and compared. The humoral IgG responses showed that the MBVax and CCVax candidates, which were more robustly elicited in all CCVax groups than in MBVax animals, improved the IgG endpoint titers dose-dependent manner. Following the second week of the most recent vaccination, it was discovered that the CCVax groups had greater fold increases in neutralizing Ab levels, ranging from 2 to 7.6-fold. In both vaccine-receiving groups, the neutralization titers peaked four months after vaccination; nevertheless, by the end of the first year, they remained similar. All of the recorded time points showed increased IgG and neutralizing Ab titers due to the CCVax formulations. Researchers demonstrated that, compared to mouse-brain-derived vaccinations, dose-dependent administration of cell-culture-purified and formalin-inactivated vaccine candidates produced robust protection in vaccinated mice [39].

Bulgaria employs a vaccine derived from the inactivated brain of a neonatal rodent to protect CCHF. At this time, strain V42/81 is being utilized to prepare the vaccine. Given its significant contribution to the immune response, the complete M-segment sequence of the V42/81 strain was deduced. Significant genetic diversity was identified within the CCHFV subtypes. To obtain a better understanding of the strain’s topology in the CCHFV phylogenetic trees, additional sequencing and analysis were performed on the complete S and fragmentary L segments [213]. To disrupt IFN-I signaling in immunologically intact, wild-type mice after CCHFV infection, mAb-5A3 was delivered in this work using the transiently immune-suppressed (IS) animal paradigm. Investigators aimed to evaluate the immunological response and efficacy of inactivated vaccines produced from mouse brain and cell culture against CCHFV. While both vaccine formulations have shown complete protection, the vaccination generated from cell culture has proven more effective in inducing T-cell responses and Ab specific to CCFHV. This work is the first assessment of the immunological response and efficacy of vaccines produced from mouse brain and cell culture in the IS mouse CCHFV model [214].

Researchers offer the initial comprehensive examination of the cellular and humoral immune reactions in healthy individuals after receiving the inactivated Bulgarian vaccine, which is presently the sole CCHFV vaccine accessible. Vaccinated individuals exhibited a significant increase in anti-CCHFV-specific T-cell activity, as determined by the IFN-γ ELISpot assay. The incidence of T-cells secreting IFN-γ was sensibly greater in individuals who received four doses of vaccination than those who received a single dose. After the initial dose, significant quantities of CCHFV Abs were detected; however, it took multiple doses to induce Ab with neutralizing activity against CCHFV. The neutralizing activity, however, was minimal in these groups. The Bulgarian vaccine elicits a T-cell response specific to anti-CCHFV. Ab produced in response to this vaccine by immunized individuals has minimal neutralizing activities. Diverse vaccine dosages elicit notably specific immune responses directed against CCHFV [215].

### Multiepitope Vaccine against CCHF

Using a computer-aided vaccine design methodology, Nosrati et al. created the first multi-epitope recombinant vaccine for CCHF in this study. To accomplish this, linear epitopes for B-cell and T-cell binding were predicted from two structural GPs of the CCHF virus, namely Gc and Gn. The antigenicity, allergenicity, hydrophobicity, stability, toxicity, and population coverage of the epitopes were investigated further. In evaluating seven epitopes in total, five of which were T-cell and two of which were B-cell, were considered for inclusion in the final vaccine construct. The final vaccine construct consisted of 382 amino acid residues, systematically arranged into four domains. These domains contained adjuvants such as heat-labile enterotoxin IIc B subunit (LT-IIc) and linear B-cell and T-cell epitopes. The segments were all connected utilizing suitable linkers. An assessment was conducted on the physicochemical properties and IFN-γ-inducing epitope content of the proposed vaccine to ascertain its solubility, stability, and capacity to elicit cell-mediated immune responses. Molecular docking experiments involving MHC-I and II molecules and the prediction of computational B-cell epitopes were performed on the three-dimensional structure of the proposed vaccine. In addition, molecular dynamics (MD) simulations were conducted to examine the stability of vaccine-MHC complexes throughout the stimulation period. The findings indicate that the vaccine we put forth exhibited stability, high solubility in water, and potential antigenicity. Furthermore, the vaccine induced both humoral and cell-mediated immune responses, suggesting that it may function as a promising candidate for an anti-CCHF vaccine [216].

The glycoprotein, NP, and RNA-dependent RNA polymerase of CCHF were used to identify the most crucial T- and B-cell epitopes for immune response. Following that, a comprehensive computational vaccinology method was used to develop a vaccine candidate consisting of many epitopes targeting the virus. A multiepitope vaccine was developed after thorough evaluation, exhibiting antigenicity, immunogenicity, and non-allergenicity while possessing the necessary physicochemical characteristics. The vaccine-receptor combination exhibits robust stability in MD simulations, indicating the resilience of the vaccine candidates against the specific immunological receptor they target. Furthermore, the immunological simulation of the vaccine candidates revealed that the vaccine has the potential to induce immune responses in people that closely resemble those seen in real-life situations. In conclusion, researchers determined that the developed multiepitope vaccine candidates will provide exceptional preventive characteristics against CCHF [217].

Five B-cells and two each of the MHC-II (HTL) and MHC-I (CTL) epitopes were selected from the two structural GP (Gc and Gn in the M segment) of CCHFV, which also includes an N-terminus EAAAK sequence and human β-defensin as an adjuvant. An exhaustive examination was conducted on the antigenicity, allergenicity, IFN gamma induction, anti-inflammatory responses, stability, and toxicity of the epitopes. The MD simulation was employed to ascertain the binding complexes’ potency by connecting the vaccine with TLR-3, TLR-8, and TLR-9 receptors and predicting its three-dimensional structure. The subunit vaccine construct was generated using the pDual-GC plasmid after codon adaptation. Currently, this structure offers population coverage encompassing 98.47% of the worldwide populace (combined HLA-I and II). The immune simulation experiments were carried out utilizing the in-silico interface C-ImmSim. The findings revealed a substantial upregulation in the production of IL-2, TGF-β, IL-10, and IL-12, as well as cellular and humoral reactions (specifically B-cell and T-cell). The researchers indicated that the suggested vaccine can elicit humoral and cell-mediated immune responses. Therefore, it emerges as an innovative and captivating contender for a vaccine targeting CCHFV [5] (Table 4).

**Table 4:** Several CCHFV vaccine strategies. The CCHFV vaccination strategies are included in this table according to the platform technology that was used to generate them: mRNA vaccines, virus-like vaccines, inactivated pathogen vaccines, replication-defective viral vector vaccines, protein subunit vaccines, and multi-epitope recombinant vaccines. CCHFV would be protected by creating a vaccine to stop infection in at-risk human populations.

Vaccine Name	Vaccine platform	Results	Ref.
vaccine-MHC	Multi-epitope recombinant vaccine	The vaccine we put forth exhibited stability, high solubility in water, and potential antigenicity. Furthermore, the vaccine-induced both humoral and cell-mediated immune responses, suggesting that it may function as a promising candidate for an anti-CCHF vaccine.	[216]
Bulgarian vaccine	Inactivated vaccines	The Bulgarian vaccine elicits a T-cell response specific to anti-CCHFV. Antibodies produced in response to this vaccine by immunized individuals have minimal neutralizing activities.	[217]
CCVax and MBVax vaccines	Inactivated vaccines	The humoral IgG responses showed that the MBVax and CCVax candidates, which were more robustly elicited in all CCVax groups than in MBVax animals, improved the IgG endpoint titers dose-dependent manner.	[39]
Experimental vaccines	Recombinant vesicular stomatitis viruses (rVSV) vaccine	The vector was well tolerated and exhibited complete efficacy when tested against a clinical strain of CCHFV. Titers of neutralizing antibodies and anti-CCHFV-GP IgG were detected in surviving animals.	[207]
CCHF VRPs vaccine	vaccine comprised of CCHF virus-like replicon particles (VRP)	The administration of VRPs, which resemble the composition and structure of genuine CCHFV, could potentially elicit a more pronounced establishment of CCHFV immunity compared to existing experimental vaccine alternatives.	[206]
ChAdOx2 CCHF vaccine	Chimpanzee adenoviral vectored vaccine	The administration of the adenoviral vaccine in combination with the Modified Vaccinia Ankara vaccine (MVA CCHF) induces the highest levels of cell-mediated and antibody responses specific to CCHFV in mice.	[205]
Ad-based vaccine	Based on human adenovirus type 5 (Ad) vaccine	Findings on the limited protective effectiveness of an Ad-based vaccine vector carrying the CCHFV N (Ad-N) against a lethal challenge of CCHFV in a mouse model that is immunocompromised and highly susceptible.	[192]
MVA-NP vaccine	Attenuated poxvirus vector vaccine	When challenged with the CCHF virus, the MVA-NP vaccine candidate showed antigen-specific immunogenicity in mice but no protective benefits. This indicates that vaccinations against the CCHF virus must show protection in a fatal dosage scenario before protective effectiveness can be demonstrated since there are no immunological correlates of protection.	[204]
CCHF_GN728 vaccine	mRNA-based multi-epitope vaccine	Optimized codons guaranteed the mRNA was expressed efficiently in the host cell. The vaccination demonstrated consistent and robust interactions with the Toll-like receptors. The researchers’ results indicate that the CCHF_GN728 vaccine will elicit targeted immune responses against the CCHFV virus.	[203]
CCHFV-MAfg09 vaccine	DNA vaccine	Demonstrates that the protective effectiveness of the M-segment-based DNA vaccine against CCHFV depends on the CD8 + T-cell response. Since antigenic strategies to target CD8 + T cells differ from a global immune system strategy, the insights garnered from these studies could be utilized to enhance the design of the GPC component of CCHFV vaccines.	[197]
mRNA vaccine	Nucleic acid vaccines	The results of the immunological tests, such as intracellular IFN-gamma staining, in-house sandwich ELISA, IL-4 and IFN-gamma ELISPOT, and survival data, amply demonstrated that their construct induced the production of immune responses directed against the nucleocapsid (N) in both mouse models.	[202]
CCHFV mRNA-LNP vaccine	Nucleic acid vaccines	Research findings indicate that the use of viral nucleotides or GP in a CCHFV mRNA-LNP vaccine provides protection against illness caused by CCHFV. Therefore, genetic immunization emerges as a persuasive approach to prevent illnesses induced by CCHFV.	[201]
DNA-based vaccine	Nucleic acid vaccines	Show that in cynomolgus macaques, plasmid-expressed CCHFV NP and GPC antigens mainly stimulate humoral and cellular immunity, respectively. Their results indicate that a two-dose vaccination strategy with plasmids expressing the NP and GPC offers significant protection against CCHFV infection.	[196]
DNA-based vaccine	Nucleic acid vaccines	The vaccine was composed of two plasmids, each encoding the NP and GPC of CCHFV. They observed robust T-cell and antibody responses in animals that had received three vaccinations. The NP + GPC vaccinated animals were considerably more resistant to CCHF-induced disease, viremia, and high tissue viral burdens than the sham-vaccinated animals.	[195]
CCHFV-Afg09-2990 M-segment DNA vaccine	Nucleic acid vaccines	Document full immunity against the CCHFV-Afg09-2990 challenge after administering a CCHFV-Afg09-2990 M-segment DNA vaccine. In conclusion, Suschak et al. demonstrate that GP38, a non-structural M-segment protein, exerts an impact on the immunogenicity of the CCHF vaccine and confers substantial protection against homologous CCHFV challenge.	[10]
Replicating RNA vaccine	Nucleic acid vaccines	Rhesus macaques immunized with RNAs encoding the CCHFV NP and GPC exhibited considerable protection against the CCHFV challenge and developed robust non-neutralizing humoral immunity against the CCHFV NP.	[194]
pDual-GC plasmid subunit vaccine	Multiepitope vaccine	The C-ImmSim in-silico interface was used to conduct the immunological simulation studies. The results showed a significant increase of humoral and cellular responses, as well as the production of IL-2, TGF-β, IL-10, and IL-12 (particularly B-cell and T-cell). The proposed vaccination may induce humoral and cell-mediated immune responses, according to the researchers.	[5]

### CCHFV Vaccination Limitations and Advantages

Historically, animal models for CCHFV have been constrained by the absence of appropriate animal hosts. Prior to identifying CCHFV animal models, efforts to develop a vaccine against CCHF were limited, and efficacy studies were unattainable. In recent years, however, scientists have created animal models that replicate the disease in humans with greater precision, offering a more precise depiction of the virus. In addition, recent developments in biochemical and molecular techniques have made it possible for scientists to develop CCHFV vaccine candidates using a variety of vaccine platforms. Preclinical investigations into vaccines for diverse platforms have yielded encouraging outcomes. However, the genetic variability of CCHFV complicates the development of a vaccine capable of conferring broad protection against all virus strains. In addition, most CCHF vaccine research has utilized the prototype IbAr10200 CCHFV strain, which was isolated from ticks and whose virulence in humans is unknown. Heterologous challenge studies are necessary to develop more dependable vaccines that offer comprehensive protection against various strains of the CCHFV to address these challenges. Despite promising preclinical investigations, the efficacy of these vaccines in human trials is yet to be determined; thus, the applicability of these findings in human clinical trials is uncertain [40].

Notwithstanding the genetic heterogeneity observed in the M segment of CCHFV, neutralizing epitopes exhibited conservation across all examined strains. Consequently, vaccines containing a standard M segment GPC have the potential to protect against various virus strains without requiring additional insert antigen modification. Immunoinformatics and in silico molecular interaction have also been applied to predict epitopes on the RNA-dependent RNA polymerase encoding the L segment and the CCHFV NP. Protection has been demonstrated in preclinical studies with vaccines containing CCHFV NP, GPC, or only GP Gn and Gc, indicating that CCHFV, includes a vast array of protective epitopes. Without immunoinformatic studies, however, the identification of specific protective epitopes within these antigens is challenging. Although creating an epitopic vaccine may offer regulated and effective immune responses, thereby mitigating the adverse effects of live vaccination, it is not without its drawbacks: extreme HLA polymorphism further complicates the consideration of epitope binding with HLA. Furthermore, although many of the characterized potential epitopes pertain to neutralization activity, the crucial role played by the T cell immune system in providing protection is frequently disregarded [218, 219].

Neutralizing Ab levels have not yet been linked to vaccine-mediated protection against CCHFV via a mechanism that has been established. It is worth noting that vaccines based on mRNA and DNA have demonstrated substantial protection in rodents or NHPs, as they do not produce detectable quantities of neutralizing Ab after vaccination. A lethal dose challenge study revealed that vaccines composed of subunits and VLPs were ineffective in protecting rodents, notwithstanding the induction of substantial quantities of neutralizing Ab. The data mentioned above indicate that while humoral immunity is significant, neutralizing Ab does not serve as a prerequisite or sufficient to provide vaccine-mediated protection against CCHFV [220–222].

Unlike the worldwide effect of SARS-CoV-2, research endeavors aimed at creating a vaccine for CCHFV are mainly concentrated on specific regions due to the belief that this virus mainly affects the Middle East, as well as some nations in Asia and Africa. While the virus was first discovered in the 1940s, its recognition as a hazardous infectious illness capable of causing a significant epidemic only occurred in 2015. This feature adds to the virus’s persistent novelty and lack of attention within the scientific community. Moreover, the predominant body of research on CCHFV is carried out by research teams associated with institutions located in locations where the virus is widespread or as part of research initiatives like the Horizon project of the European Union, which limits worldwide participation. Although the virus is currently limited to a specific geographic area, it is crucial to establish and maintain long-term, collaborative studies involving multiple institutions and disciplines. These studies should integrate immunoinformatic tools with in vivo and in vitro research to investigate the virus’s molecular structure, pathophysiology, immunological properties, and other related aspects. The aforementioned situations and characteristics contribute to the limitations associated with producing vaccines for CCHFV and provide possible directions for future research in this field [233, 223].

## Future and Landscape

The WHO has designated CCHF as a high-priority pathogen because of its significant fatality rate. This study was carried out in the county that exhibited the highest incidence of CCHF cases, the province that encompassed the largest high-risk area for its occurrence, and the country that maintains the highest incidence of CCHF globally. While prior research has examined the KAP of healthcare personnel and butchers in Iran, this is the first study to explore the KAP of Iranian livestock producers residing in a community with a high CCHF burden. The present study revealed that the mean scores for overall knowledge and behavior were deemed unacceptable. A One-Health approach, which includes biosurveillance of humans, animals, and parasites, is crucial in this circumstance to effectively monitor and prevent human outbreaks [57]. WHO places CCHFV in WHO Risk Group IV, which means that any research involving infectious viruses is advised to utilize maximal biocontainment facilities. Due to the lack of effective prophylactic or therapeutic regimens, CCHFV is categorized as a Select Agent, a Risk Group 4 Pathogen, and an NIAID Category C Priority Pathogen in the United States. Funds for investigating CCHFV have been made available to researchers due to its Priority Pathogen classification; however, access to the pathogen has been restricted due to the Biosafety Level 4 working condition requirement. The CCHFV is regarded as a possible biological weapon. Furthermore, the lack of appropriate animal models and the WHO Risk Group IV classification of CCHFV has impeded the development of new preventative and therapeutic measures. Although ribavirin has demonstrated activity against CCHFV in vitro, clinical studies have yet to establish its efficacy as a treatment for humans definitively. CCHF-immunoglobulin is also utilized; however, its efficacy remains unsubstantiated [36]. The worldwide appearance and reappearance of CCHF underscore the necessity for additional foundational efforts to regulate the pathogen, which presents significant risks to the well-being of both humans and animals. Developing countermeasures, treatment options, and immunocompetent CCHF disease models must be the primary focus of future research. Furthermore, apart from the absence of research, the data unveiled a significant knowledge deficit about the implementation of personal protective measures. Hence, it is imperative to commence educational initiatives targeting individuals involved in hazardous occupations, including abattoir workers, agricultural laborers, veterinarians, and herdsmen [1].

The identification of a virus is determined by its detection and separation. Molecular and serological approaches may be used to identify the virus in the early stages of infection. One of the procedures used is RT-PCR, a molecular method capable of identifying several genetic lineages of CCHFV, both local and non-local. ELISA and IFA are serological techniques used to identify IgM and IgG Ab generated as a result of an infection. Ab may be determined five days after infection. Viruses may be obtained from the bloodstream when there is a large concentration of viruses in the pre-hemorrhagic phase. The isolation of CCHFV involves using certain cell lines, including BHK-21, LLC-MK2, Vero, SW-13, CER, Huh7, and HepG2. CCHFV is an infectious illness. Hence, the identification and segregation of the virus must be conducted inside a biosafety level 4 (BSL-4) containment laboratory by trained professionals to avoid any untoward incidents [224]. In regions afflicted with CCHF where PCR and serological assays are delayed, early detection may depend critically on the knowledge of predictive parameters [225].

When VHF of unknown etiology is introduced, it is anticipated that the multiplex virus test method devised in this study will be utilized for quarantine and public health [226]. . Research has successfully developed a multiplex test to detect Ab against many CCHFV antigens in a single sample, regardless of the ruminant species. This test has significant potential in surveillance studies, enabling effective monitoring of the dissemination of CCHFV and proactive prevention of further outbreaks. Additionally, it offers valuable insights into the immunological response elicited by CCHFV [227]. Rapid and simultaneous detection of multiple targets, as opposed to a single target, would therefore significantly improve medical diagnoses. In addition, it is anticipated that implementing artificial intelligence (AI) will enhance diagnostic efficacy. As a result, mass detection, prompt diagnosis, and physical interventions such as social distancing continue to be essential in preventing the spread of the virus. Therefore, the continuous development of rapid, robust, specific, and highly sensitive point-of-care diagnostic assays remains imperative. Given the criteria mentioned above, the purpose of this review is to present a comprehensive outline of the current diagnostic methodologies employed in the context of CCHFV infection, with a particular emphasis on their ramifications for public health and emergency response. The gold standard for conventional CCFHV diagnosis is the utilization of RT-PCR for nucleic acid detection. RNA extraction is a laborious process that necessitates the presence of qualified technicians and costly laboratory facilities. The extraction process itself can take up to four hours. While RT-PCR is the principal method used to combat the outbreak, alternative serological diagnostic techniques are also available to supplement it. Cross-reactivity and the possibility of erroneous positive results are, however, substantial causes for concern. In light of the ongoing transmission of the disease, there is an urgent need for more cost-effective point-of-care instruments. Implementing biosensors and microfluidics technology has significantly accelerated the development of health monitoring systems by enabling rapid detection methods. Microfluidic technology allows the incorporation of intricate analytical procedures into minute volumes, including but not limited to temperature regulation, sampling, combining, separation, enrichment, and cleansing [228–230]. Regarding the early detection of CCHFV infection, novel diagnostic approaches based on biosensors and nano biosensors have received scant consideration. Although knowledge and research gained from other virus investigations, such as COVID-19, can be applied to detecting CCHFV biomarkers using nanoparticles.

The mainstay of treatment for CCHF consists of supportive measures. Optimal care should encompass vigilant monitoring of fluid equilibrium and rectification of electrolyte imbalances, provision of oxygenation and hemodynamic support, and suitable management of secondary infections. In vitro, the virus exhibits susceptibility to the antiviral medication ribavirin [231]. The recent use of mesenchymal stem cells (MSCs) to treat acute respiratory distress in a subgroup of COVID-19 patients has generated prospective benefits for using MSCs as a supportive therapy for this viral pathogen in elderly patients with severe pneumonia and patients with acute conditions. The therapeutic effectiveness and safety of exosomes (EXOs) in transporting various cellular biological components to the recipient cell have garnered considerable interest due to their capacity to transport miRNAs. MSCs can produce a diverse array of EXOs (MSC-EXOs), indicating that they may be clinically viable alternatives to other cell origins for EXO generation. MSC-EXOs have been the subject of extensive research due to their tumor-homing capabilities, adaptability, and immune attributes. Additionally, several initial findings that validate the potential of MSCs as a viable therapeutic option for controlling this infection were delineated. The application of MSCs to treat COVID-19-associated ARDS is highly experimental. Patients with severe pneumonia caused by COVID-19 who received MSC infusions reported an improvement in respiratory function from the protection of epithelial and endothelial cells and an unidentified modulation of hypercytokinemia. Profound preclinical efficacy data, favorable clinical trial outcomes involving MSCs in patients with COVID-19, and the established safety profile of MSCs collectively demonstrate their therapeutic potential for SARS-CoV-2 patients presenting with severe manifestations and hyper-inflammatory conditions [151, 232, 233]. Furthermore, novel therapeutic approaches, including stem cell therapy and its derivatives, have received negligible consideration when it comes to the management of CCHFV infection. However, we can utilize the knowledge and research that has been conducted on other viruses to aid in treating this illness.

Significant advancements are now being achieved in developing a vaccine for CCHFV, facilitated by the existence of a very effective animal model for studying the deadly virus. The emergence of the EBOV in West Africa in 2014 has drawn significant focus to VHFs. As various vaccines for filoviruses are currently being developed, it is anticipated that attention will shift toward other pathogens that cause VHFs, such as CCHFV. The newly released WHO R&D Blue Print emphasizes the responsibility of international governments and non-profit entities to finance the development and accumulation of novel vaccines for emerging high-consequence infections. The Coalition of Epidemic Preparedness Innovations (CEPI) was founded to invest and advance the development of novel vaccines for emerging diseases. It received an initial financing of $500 M from the World Economic Forum and several foreign governments. The UK Government is actively financing the development of a protective vaccination based on the MVA viral vector backdrop. Human clinical trials for this vaccine will commence in late 2017. To be deemed safe and readily accessible for emergency immunizations, CCHF vaccines must undergo early human clinical studies. Recent breakthroughs in this sector provide optimism that, in the future, CCHFV may be effectively managed in regions where it is prevalent and during epidemic situations [233]. In addition, numerous vaccine studies have been conducted in recent years; however, safety concerns, high toxicity, and inadequate protection against strain sequence variability have resulted in the absence of licensed vaccines against CCHFV infection. The application of computational immunology and vaccine informatics methodologies to develop a multi-epitope vaccine devoid of undesirable, toxic, and allergic peptide fragments is becoming more prevalent. It is now routinely employed before experimental vaccinology. These methodologies are effectively implemented against various infectious, viral, and bacterial pathogens. Immunoinformatics’ primary aim is to identify antigenic, safe, and immunodominant epitopes that elicit robust and risk-free immune responses against the pathogen and satisfy all criteria for vaccine candidacy. Because CCHFV is linked to substantially increased morbidity and mortality globally, computational studies are required to develop a fictitious vaccine construct that can be readily evaluated in subsequent experiments for protection against CCHFV [234].

## Conclusion

Early detection of CCHFV is essential for good patient management and the avoidance of possible transfer to care workers caring for the patient. CCHFV diagnosis is essentially difficult, whether in clinical check or laboratory evaluation, owing to the unique features of each collection of signs, which may or may not be present, the likenesses among one or more signs, and the phylogenetic cross-reaction in serological trials that frequently occurs, particularly in endemic areas. Most arbovirus diagnostic tests have a high level of specificity, which is crucial in the differential diagnosis of these rapidly spreading viruses. Many approaches and tactics have been offered to improve and differentiate these viruses, including from standard molecular diagnostic assays, which are backed by the excellent reliability of rt–PCR, nested RT–PCR, and RT–PCR machines, to POC immunochromatographic tests and new biosensors. Lastly, molecular identification is only feasible in the severe stage of the disease, which lasts just a few days after infection. Serological assays, on the other hand, frequently fail to distinguish Ab from viruses of the identical genus, especially IgG. Given the significant pathogenicity of CCHFV and the substantial risk of human-to-human transmission, it is crucial to devise innovative detection techniques that are as follows: rapid, dependable, easily obtainable, cost-effective, secure, and highly sensitive diagnostic instruments. Nevertheless, further investigation is warranted in this domain. In addition, it is necessary to educate the general public, healthcare professionals, and cultivators regarding the causes, transmission, and dangers of CCHFV. Developing a surveillance system, standard preventative measures, early detection, appropriate treatment, and rapid response necessitates an immediate course of action. Without such a strategy, the buildup of components accountable for the unforeseen proliferation of CCHFV could pose a significant threat to human life throughout the nation.

At now, supportive therapy is the primary method of treatment. Ribavirin, a medicine that can combat a wide range of viruses, has been given to individuals with CCHF. However, the effectiveness of this treatment is yet uncertain. Additional treatment options in case studies include corticosteroids, convalescent serum, and targeted immunoglobulin. However, there is currently little information to evaluate the efficacy of these medications. Furthermore, researchers have assessed prospective substances that might hinder the activity of bunyaviruses for many years. Some of these substances have shown promising effectiveness in combating CCHFV infection. Due to several impediments to advancement, CCHF will continue to pose a substantial risk to public health in the foreseeable future. Nevertheless, to progress a promising candidate for therapeutic use, it will be imperative to conduct investigations in bigger animal models that accurately replicate human ailments. Although existing medicines for CCHFV have been evaluated using restricted animal models and clinical trials, future efforts should prioritize the development of more suitable animal models for pre-clinical studies on medications.

Parallel to the development of novel molecular biology techniques and the establishment of minuscule animal models susceptible to CCHFV, our comprehension of the structure and function of CCHFV viral proteins continues to progress. Ongoing research is being conducted to investigate the mechanisms of illness and host selection of CCHFV. In particular, an enhanced comprehension of the correlation between the protective efficacy of CCHFV vaccines and the quantities of neutralizing Ab detected in laboratory assays could provide valuable insights for advancing innovative vaccine design methodologies that potentially stimulate more robust immune responses. Additional research across multiple disciplines—including molecular biology, virology, veterinary medicine, bioinformatics, and protein engineering—is required to address these developments. Vaccines developed using conventional methods show improved effectiveness when evaluated on the immune systems of model species. However, they do not achieve the desired outcomes when given to people due to the complex nature of the human immune system. Therefore, by using reliable reverse vaccinology, vaccine informatics, and biophysics techniques, this scientific study produced a vaccine candidate that is not only safe but also highly productive, targeted, and durable against CCHFV. Given the poor understanding of early identification, treatment, and prevention, future research must focus on developing therapies to mitigate the increasing risk of CCHF. Several gaps in understanding have been discovered regarding the molecular basis and treatment of CCHFV infection. Therefore, future research should prioritize these areas.

## Data Availability

No datasets were generated or analysed during the current study.
